# A maternal dorsoventral prepattern revealed by an asymmetric distribution of ventralizing molecules before fertilization in *Xenopus laevis*

**DOI:** 10.3389/fcell.2024.1365705

**Published:** 2024-03-20

**Authors:** Aitana M. Castro Colabianchi, Nicolás G. González Pérez, Lucía F. Franchini, Silvia L. López

**Affiliations:** ^1^ Universidad de Buenos Aires, Facultad de Medicina, Departamento de Biología Celular e Histología / 1° U.A. Departamento de Histología, Embriología, Biología Celular y Genética, Laboratorio de Embriología Molecular “Prof. Dr. Andrés E. Carrasco”, Buenos Aires, Argentina; ^2^ CONICET–Universidad de Buenos Aires, Instituto de Biología Celular y Neurociencias “Prof. E. De Robertis” (IBCN), Buenos Aires, Argentina; ^3^ Instituto de Investigaciones en Ingeniería Genética y Biología Molecular (INGEBI) “Dr. Héctor N. Torres”, Consejo Nacional de Investigaciones Científicas y Técnicas (CONICET), Buenos Aires, Argentina

**Keywords:** bilateral symmetry, axialization, maternal determinants, maternal asymmetries, bilaterians, notch

## Abstract

The establishment of the embryonic dorsoventral axis in *Xenopus* occurs when the radial symmetry around the egg’s animal-vegetal axis is broken to give rise to the typical symmetry of Bilaterians. We have previously shown that the Notch1 protein is ventrally enriched during early embryogenesis in *Xenopus laevis* and zebrafish and exerts ventralizing activity through β-Catenin destabilization and the positive regulation of ventral center genes in *X. laevis*. These findings led us to further investigate when these asymmetries arise. In this work, we show that the asymmetrical distribution of Notch1 protein and mRNA precedes cortical rotation and even fertilization in *X. laevis.* Moreover, we found that in unfertilized eggs transcripts encoded by the ventralizing gene *bmp4* are also asymmetrically distributed in the animal hemisphere and *notch1* transcripts accumulate consistently on the same side of the eccentric maturation point. Strikingly, a Notch1 asymmetry orthogonal to the animal-vegetal axis appears during *X. laevis* oogenesis. Thus, we show for the first time a maternal bias in the distribution of molecules that are later involved in ventral patterning during embryonic axialization, strongly supporting the hypothesis of a dorsoventral prepattern or intrinsic bilaterality of *Xenopus* eggs before fertilization.

## Introduction

**FIGURE 1:**
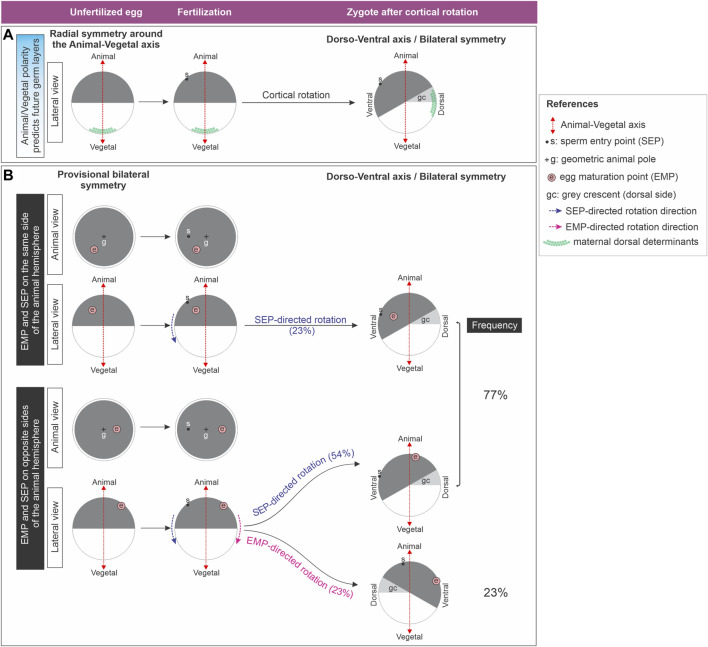
**(A)** Classic model which considers that the *Xenopus* unfertilized egg is radially symmetric around the animal-vegetal axis. Radial symmetry is broken shortly after fertilization by cortical rotation through relocation of maternal dorsal determinants from the vegetal pole to the future dorsal side. **(B)** *Xenopus* unfertilized eggs bear an intrinsic bilateral symmetry related to the position of the eccentric EMP, which can direct dorsoventral axialization if the SEP-oriented cues fail. Diagram representing the results obtained by Brown et al. (1994) (Brown et al., 1994), who analyzed the direction of yolk mass rotation in eggs with the cortex artificially immobilized, here illustrated as cortical rotation for simplicity. Only in eggs in which the sperm entered the animal hemisphere on the opposite side of the EMP, the later can override the SEP to direct rotation. Global frequencies for either SEP- or EMP- directed rotation were calculated from the results presented on Figure 6 of (Brown et al., 1994) and are shown as percentage of the total number of eggs analyzed (n = 31) in the right column. From the same dataset we also calculated the frequencies for SEP- or EMP- directed rotation for the eggs with the EMP and SEP on the same or on opposite sides of the animal hemisphere (percentages between brackets).

Amphibian eggs bear an animal-vegetal polarity that roughly predicts the future array of presumptive germ layers, with germ cell determinants accumulating in the vegetal pole from the earliest stages of oogenesis (Dale and Slack, 1987; Moody, 1987; Card, 1995; Houston, 2022). So far, it was described that before fertilization in *Xenopus*, maternal asymmetries in the distribution of transcripts only exist along the animal-vegetal axis (Figure 1A, left). They appear during oogenesis (Kloc and Etkin, 1995; Kloc et al., 2001; Sindelka et al., 2018) and are also present during early cleavage stages (De Domenico et al., 2015). This radial symmetry around the animal-vegetal axis (Figure 1A, left) is considered to be broken shortly after fertilization because the embryonic dorsoventral axis is established on the meridian passing through the animal pole and the site where the gastrula organizer (the dorsal blastopore lip) will appear (Figure 1A, right). Since this initial dorsoventral axis sets the position of the future body midline, the embryo acquires the typical symmetry of Bilaterians relative to the plane of bilateral symmetry, with the concomitant emergence of anterior-posterior and mediolateral dimensions (Gerhart et al., 1989; Ubbels, 1997). In *Xenopus*, this dorsoventral polarity is generated by cortical rotation, a process normally triggered by fertilization, with the sperm entering the egg’s animal hemisphere (Figure 1A). Cortical rotation starts around the mid-first cell cycle and concludes shortly before first cleavage, shifting the zygote’s vegetal cortex in relation to the cytoplasmic core 30° away from the sperm entry point (SEP) towards the future dorsal side (Gerhart et al., 1989) (Figure 1A). If the egg’s cortex is artificially immobilized, the cytoplasmic core, along with the yolk mass, rotates towards the SEP, also relocating the vegetal cortex to the future dorsal side (Gerhart et al., 1989; Brown et al., 1994).

Maternal dorsal determinants like *wnt11b* mRNA and Dishevelled protein move during this rotation from the vegetal pole to the future dorsal side (Figure 1A). During early cleavage stages, Wnt11b activates Dishevelled, leading to Glycogen synthase kinase-3 (GSK3) degradation dorsally and β-Catenin stabilization. Then, nuclearized β-Catenin activates dorsal genes, whereas in the ventral side, GSK3 continues tagging β-Catenin for degradation in a destruction complex involving Axin1 (Molenaar et al., 1996; Miller et al., 1999; Wessely et al., 2001; Weaver et al., 2003; Weaver and Kimelman, 2004; Wessely et al., 2004; Tao et al., 2005; Ishibashi et al., 2008; Verheyen and Gottardi, 2010; Houston, 2017; Tassan et al., 2017). β-Catenin’s dorsal stabilization by a Wnt ligand is nowadays debated, and it was reported that maternal Wnt11b rather is required for robust cortical rotation (Houston, 2022; Houston et al., 2022). The maternal mRNA *huluwa* (*hwa*) is vegetally deposited in *Xenopus* oocytes and becomes dorsally enriched in early cleavage embryos. In zebrafish, it moves from the vegetal pole towards the animal region along one side of the yolk cell during early cleavage. Hwa protein localizes dorsally and promotes Axin1 degradation, stabilizing β-Catenin. It is essential for dorsal development in both species, operating independently of a Wnt ligand (Yan et al., 2018). Lysosome activation on the dorsal side is crucial for dorsal axial development in *Xenopus* (Tejeda-Muñoz and De Robertis, 2022). It was proposed that, in the ventral region, Hwa protein is ubiquitinated and undergoes lysosomal degradation, whereas dorsally, non-ubiquitinated Hwa requires intact lysosomal trafficking for its dorsalizing activity (Zhu et al., 2021; Tejeda-Muñoz and De Robertis, 2022).

Strikingly, in approximately 30% of cases, the dorsal blastopore lip does not appear opposite to the SEP side in *Xenopus*. The most accurate predictor for the position of the future dorsoventral axis is the direction of cortical rotation, which can occur in the absence of sperm, after activating the egg by parthenogenesis. The microtubules of the sperm aster are not necessary for cortical rotation, but they polarize its direction (Black and Vincent, 1988; Gerhart et al., 1989; Ubbels, 1997; Tassan et al., 2017). Moreover, several amphibian eggs have some degree of latent dorsoventral asymmetry which reveals signs of bilateral symmetry. Some authors consider that the assumption that unfertilized amphibian eggs have perfect radial symmetry around the animal-vegetal axis should be revised (Brachet, 1977; Brown et al., 1994; Ubbels, 1997). During maturation of *Xenopus* stage VI oocytes, transition to metaphase II occurs, with the breakdown of the nucleus and the extrusion of the first polar body, leaving a white spot in the pigmented animal hemisphere. This “egg maturation point” (EMP) is eccentric in relation to the geometric center or geometric animal pole (GAP) of the animal hemisphere in about 70% of the spawned eggs (Brown et al., 1994) (Figure 1B). Remarkably, an intrinsic asymmetry of the unfertilized egg, termed “provisional bilateral symmetry” by Brown et al. (Brown et al., 1994), dictates the direction of yolk mass rotation after activation by parthenogenesis. Here, the EMP serves as a reliable positional predictor, because the yolk mass consistently rotates towards the EMP side. Since in most fertilized eggs the yolk mass rotates towards the SEP side, the sperm generally overrides the egg’s provisional bilateral symmetry, providing a new directional cue. Strikingly, this is not always the case. For unknown reasons, some fertilized eggs (23% in the study of Brown et al.) (Brown et al., 1994) escape the directionality imposed by the sperm, and the yolk mass rotates guided by their intrinsic asymmetry. Notably, this EMP-directed rotation can only occur if the EMP and the SEP are on opposite sides (Brown et al., 1994) (Figure 1B). Thus, the *Xenopus* unfertilized egg bears an intrinsic bilateral symmetry which can direct dorsoventral axialization if the SEP-oriented cues fail. The molecular basis underlying this intrinsic bilateral symmetry is unknown.

The embryo’s dorsal center, induced by β-Catenin, acts in concert with a ventral center to regulate the dorsoventral and anterior-posterior embryonic axes. Their reciprocal antagonistic relationship, extensively studied in amphibians and fish, involves the ventral center secreting BMP4 and Wnt8a morphogens to induce ventral-posterior fates. Meanwhile, the dorsal center expresses antagonists and transcriptional repressors of ventral morphogens, safeguarding the dorsal region from ventralization and posteriorization (De Robertis, 2009; Thisse and Thisse, 2015). It remained unclear if an event of asymmetric distribution of ventralizing molecules occurs at the beginning of embryogenesis. *Xenopus* transplantation experiments suggest local influences on ventral cell fates start from at least the 16-cell stage (Gallagher et al., 1991). While previous studies demonstrated the dorsalizing activity of polyA + mRNA from dorsal-animal blastomeres (Pandur et al., 2002) and dorsal accumulation of *wnt11b* transcripts at early cleavage stages (Castro Colabianchi et al., 2018; Schroeder et al., 1999; Tao et al., 2005), an enrichment of *wnt8b* mRNA in ventral-animal blastomeres at the 16-cell stage was also observed (Pandur et al., 2002). This hints at an early appearance of an asymmetric distribution of dorsally and ventrally enriched molecules. Strikingly, RNAseq analysis of total (4 embryos) or polyadenylated mRNA (1 embryo) did not find significant differences in transcriptomes between *Xenopus* dorsal and ventral cells at the 8-cell stage (De Domenico et al., 2015). However, mass spectrometry assays showed significant differences between dorsal and ventral proteomes at the 16-cell stage (Lombard-Banek et al., 2016) and in the metabolome at the 8 and 16-cell stage (Onjiko et al., 2015; Onjiko et al., 2017), with some proteins and small metabolites differentially enriched in the dorsal or the ventral side. This highlights that, from a molecular perspective, ventral is not equivalent to dorsal without dorsal molecules.

Our previous research revealed that Notch1 protein and mRNA are ventrally enriched in the animal hemisphere of 1-cell *Xenopus laevis* embryos, maintaining this asymmetry through cleavage stages. Employing gain- and loss-of-function approaches, we demonstrated that Notch1 contributes to the formation of the initial *Xenopus laevis* dorsoventral axis. This involves two ventralizing activities: 1) positive regulation of ventral center genes, mainly through the canonical pathway; 2) confining the blastula dorsal center through a non-canonical pathway, which promotes the destabilization of β-Catenin that escapes from the GSK3β-dependent degradation in the ventral side (Acosta et al., 2011; Castro Colabianchi et al., 2018). This local enrichment of Notch1, a molecule with proven ventralizing properties, represents the earliest sign of ventral development described so far in vertebrates, since it is evident from the 1-cell stage. Notably, mass spectrometry identified Nrarp (NOTCH regulated ankyrin repeat protein) as ventrally enriched in animal-ventral blastomeres of the 16-cell embryo, whereas GAPDH is homogeneously distributed, as expected for a housekeeping gene product (Lombard-Banek et al., 2016). This supports the idea that the Notch pathway is active at the ventral side of early cleaving embryos, since *nrarp* is regulated by canonical, RBPJ-dependent Notch signaling (Lamar et al., 2001).

Our previous findings raised the important question of whether ventralizing molecules are asymmetrically enriched at the same time or even earlier than maternal dorsal determinants. In this work, we aimed to establish when Notch1 asymmetry first arises in *X. laevis*. Surprisingly, we found that it is already present in the animal hemisphere prior to cortical rotation in zygotes and even in unfertilized eggs, both at the protein and mRNA levels. Moreover, *notch1* mRNA is not randomly distributed in unfertilized eggs, but consistently accumulates on the same side of the eccentric EMP. Strikingly, we found that the asymmetric distribution of Notch1 protein in an axis orthogonal to the animal-vegetal axis is already present in oocytes from the earliest stages of oogenesis. In addition, unfertilized eggs also show an asymmetric distribution of *bmp4* transcripts in the animal hemisphere. Our findings reveal a maternal bias in the distribution of molecules in the unfertilized egg that are known to control ventral patterning during embryonic axialization. This indicates that *X. laevis* eggs bear a latent, dorsoventral prepattern before fertilization, lending support for the first time at the molecular level to the hypothesis of their intrinsic bilaterality previously proposed by other authors (Brown et al., 1994; Ubbels, 1997). In addition, *notch1* asymmetry at the mRNA level is conserved in early zebrafish embryos from the zygote stage, supporting evidence of Notch pathway asymmetries during the earliest steps of development in Bilaterians.

## Materials and methods

### Animals and oocytes

Albino and wild-type *X. laevis* embryos were obtained by natural mating from adult males and females obtained from Nasco (Wisconsin), according to standard protocols (Sive et al., 2010). Adult animals were hormonally stimulated by injection of human chorionic gonadotrophin (Pregnyl, MSD, Argentina) (Sive et al., 2010). Eggs were obtained by gently squeezing hormonally stimulated females. Oocytes were donated by Dr. Karina Alleva´s laboratory and obtained from ovaries surgically removed from anesthetized pigmented *X. laevis* females. Ovaries lobes were transferred to ND96 buffer (96 mM NaCl; 2 mM KCl; 1.8 mM CaCl_2_; 1 mM MgCl_2_; 5 mM HEPES, pH 7.4) and oocytes were separated in smaller clumps with forceps. Oocyte clumps were incubated for 2 hs. with 1 mg/mL collagenase type I (GIBCO) in ND96, with gentle rotation (200 r.p.m). Every 15 min, oocytes were dispersed in the collagenase solution by gently pipetting them up and down through a wide tip Pasteur pipette. Once completely defolliculated, oocytes were extensively washed with ND96 buffer and fixed.

*X. laevis* embryos were staged according to Nieuwkoop and Faber (Nieuwkoop and Faber, 1994) and oocytes were staged according to Dumont (Dumont, 1972). *Danio rerio* (zebrafish) embryos were obtained as previously described (Kamm et al., 2013) and staged according to Kimmel et al. (Kimmel et al., 1995). The cortical rotation period in *X. laevis* spans from around 0.4 to 0.8 of the first cell cycle (Gerhart et al., 1989), corresponding approximately from 33 to 66 min post-fertilization (mpf) at 22°C–24°C, which is the temperature at which zygotes were collected.

### *In situ* hybridization and immunolocalization

*In situ* hybridization (ISH) probes for zebrafish *notch1a* (Ke et al., 2008) and *X*. *laevis bmp4* (Fainsod et al., 1994)*, hes4* (previously known as *hairy2a*) (Schmidt et al., 1995), *dll1* (previously known as *delta-1*) (Chitnis et al., 1995), *notch1* (Coffman et al., 1990)*, wnt11b* (Tada and Smith, 2000) were previously described.

For cloning *pou5f3.1* cDNA (previously known as *oct91*), total RNA was isolated from *X. laevis* embryos with TRIzol reagent (ThermoFisher, Cat. #15596018), reverse transcribed with oligodT and SuperScript II reverse transcriptase (TermoFisher, Cat. #18064014). *Pou5f3.1* cDNA was amplified with Pfx DNA polymerase (Invitrogen, Cat. #11708013) with specific PCR primers containing linkers with restriction enzyme sites for cloning. Their sequences were as follows: *pou5f3.1*-F, CGG​GAT​CCC​CGG​CAA​CTT​AGG​TAG​GAT​T (BamHI site underlined); *pou5f3.1*-R, GGA​TCG​ATC​GAA​GTC​TAG​TTG​CCT​TGG​T (ClaI site underlined). The PCR product was cut with BamHI and ClaI and inserted in pBluescript II SK+ with T4 DNA ligase. Identity was confirmed by sequencing. The plasmid was linearized with BamHI and the antisense probe was transcribed with T7 RNA polymerase.

Since it is not possible to distinguish the animal-vegetal axis in whole albino or bleached wild-type *X. laevis* oocytes, unfertilized eggs and zygotes, or in early stages of oogenesis in wild type oocytes, we adopted two strategies to determine if there are asymmetries in the distribution of Notch1 in the animal hemisphere: 1) For wild-type eggs, after fixation and rehydration, we isolated animal hemispheres by dissection through the equatorial plane, guided by the animal-vegetal pigmentation asymmetry. These animal hemispheres were further processed for ISH or Notch1 immunofluorescence as previously described, including a bleaching step to avoid quenching of the immunofluorescence signal by the pigment (Castro Colabianchi et al., 2018) (see below). 2) Albino eggs, albino zygotes, and bleached wild-type oocytes were processed for *notch1* ISH or Notch immunofluorescence combined with ISH for vegetal markers as spatial reference (*gdf1* for zygotes, *wnt11b* for eggs and oocytes), following the procedures described before (Castro Colabianchi et al., 2018).

The preparation of digoxigenin-labeled antisense RNA probes and the whole-mount ISH procedure for *Xenopus* were performed as previously described (Pizard et al., 2004), except that the proteinase K step was omitted. Double ISH was performed as previously described (López et al., 2005). Zebrafish embryos were fixed and processed for whole-mount ISH in the same conditions as *X. laevis* embryos, except that, after rehydration, they were dechorionated with sharp forceps pre-treated with 0.5 NaOH before proceeding to ISH. For immunofluorescence of endogenous Notch1 and GAPDH proteins, *X. laevis* oocytes, eggs, and embryos were fixed for 90 min with MEMPFA, transferred to Dent’s fixative (80% methanol, 20% DMSO) for 72 h at −20°C, and permeabilized with pre-hybridization solution in the same conditions as for ISH. For revealing the ISH, we used the following reagents: a combination of 5-bromo-4-chloro-3-indoxyl phosphate (BCIP, Biosynth, B-7500) with p-Nitroblue Tetrazolium Chloride (NBT, Biosynth, N-8100), which gives a purplish/blueish color; BCIP, which gives a turquoise color, or Magenta Phosphate (Biosynth, B-7550), which gives a magenta color.

For Notch1 immunofluorescence combined with ISH of vegetal markers, specimens were fixed with MEMPFA overnight at 4°C and, the following day, they were subjected to the standard ISH protocol. After stringency washings, they were washed with TBSE, TBSET, and TBSET + BSA as described (Acosta et al., 2011), and were incubated overnight at 4°C simultaneously with the anti-digoxigenin-alkaline phosphatase antibody for ISH (Roche, 11093274910, 1/2000) and the Notch1 antibody for immunofluorescence. After washing 4 times with TBSET for 45 min and 1 h with TBSET + BSA, specimens were incubated with the secondary antibodies overnight at 4°C. Then, they were washed three times with TBSET and processed for revealing the *wnt11b* or the *gdf1* ISH. The pattern of the vegetal marker was correlated with the immunofluorescence image for Notch1 of the same specimen.

For preparing samples for cryosections, animal hemispheres of wild-type eggs obtained and treated as previously described were impregnated in 15% sucrose in PBS for 30 min at room temperature and were then kept overnight in 30% sucrose in PBS at 4°C. The following day, specimens were incubated in 15% gelatin (Sigma, G9391) in PBS for 30 min at 37°C, in tubes of 2 mL capacity. Embedding was performed in the same solution, and specimens were oriented in such a way that the internal, equatorial surface of animal hemispheres was laid on the bottom. In this way, serial sections were obtained, with the first section being the most internal one. Embedded specimens were snap-frozen by immersion for 1 min in an isopropanol/dry ice bath at −40°C or below, and immediately transferred to −70°C overnight. Before sectioning, specimens were transferred to −20°C for 1 h. Sections of 20 μm thickness were obtained with a cryostat (Leica, CM 1850) and collected on positively charged glass slides (Fisherbrand, Superfrost Plus). Sections were dried overnight at room temperature. Immunofluorescence was performed in general as previously described for whole-mount specimens (Acosta et al., 2011), except that for sections, incubations were carried out with 0.2 mL of each solution per slide and the slides were covered with Parafilm. Antibody incubations were performed overnight at 4°C in a wet chamber, and the antibodies were washed four times for 30 min with 50 mL of PBS each. Slides were mounted with Vectashield (Vector Laboratories, H-1200).

The commercially available Notch1 intra rabbit polyclonal antibody (N1 intra Ab) (Abcam, ab8387) was raised against a peptide from the human intracellular domain of NOTCH1, which has 93% identity with the protein encoded by *X. laevis notch1* and was validated in *X. laevis* in previous work (Castro Colabianchi et al., 2018). Primary and secondary antibodies were diluted in blocking buffer as follows: N1 intra Ab (1/200), GAPDH (Ambion, AM4300, 1/100), anti-α-tubulin (DSHB, 12G10, 1/100), anti-rabbit IgG-Alexa 594 (Thermo Fisher Scientific, A-11012, 1/100), anti-mouse IgG F(ab′)2-Alexa Fluor 488 (Thermo Fisher Scientific, A-11017, 1/200). For double immunofluorescence for Notch1 and GAPDH or α-tubulin in cryosections, the comparison of both patterns was restricted to those eggs in which GAPDH or α-tubulin showed a regular expression along the section.

Specimens were bleached at the end of the ISH procedure or after the permeabilization step with pre-hybridization buffer during the immunofluorescence procedure. The bleaching step was performed in 1% H_2_O_2_, 5% Formamide, 0.5X SSC, over a fluorescent light source.

All specimens were photographed in a MVX10 fluorescence microscope (Olympus) equipped with a DP72 camera (Olympus). For visual consistency to facilitate comprehension for the reader, all images in the figures were oriented with the Notch1+ side to the left, except for the bleached animal hemisphere shown in animal view in Figure 4A″ and the morphometric analysis shown in Figure 5, where the ISH images were oriented following the polar coordinates criterion described below, with the EMP upwards.

Results are expressed as the number of specimens showing an asymmetric distribution of the analyzed marker in relation to the total number of specimens analyzed (n) and are indicated in the Supplementary Tables. Biological replicates represent batches of embryos, eggs, or oocytes from different females, and their number (N) is indicated in the Supplementary Tables. In the case of double ISH or immunofluorescence combined with ISH with the reference markers *gdf1* or *wnt11b*, analysis of the other markers was restricted to those specimens showing a vegetal signal for the reference markers.

### Image analysis for Notch1 immunofluorescence in unfertilized eggs

Notch1 and GAPDH immunofluorescence was quantified in cryosections of animal hemispheres from 15 independent unfertilized eggs (n = 15). Measurements were performed with ImageJ by defining two circular regions of interest (ROI), one on the region of visually maximum Notch1 immunofluorescence intensity (ROIa) and the other, on the opposite side (ROIb), for both, Notch1 and GAPDH immunofluorescence (Figure 3D). These ROIs comprised a portion of the egg’s membrane (where Notch1 immunofluorescence intensity is visually highest) and the adjacent cytoplasmatic area (Figure 3D). We measured the gray value per pixel, which quantifies how far from the darkness (lack of fluorescence) the measured pixels are. Therefore, this variable represents the fluorescence intensity per pixel and indirectly represents the “protein level”. For each ROI, we computed the mean pixel intensity (mpi). Statistical analysis was carried out using GraphPad Prism. As each sample always comprised a pair of measurements (as defined by ROIa and ROIb), paired t-tests (two-tailed) were performed; the difference between means was considered statistically significant when *p* < 0.05.

### Morphometric analysis of the spatial relationship between *notch1* mRNA distribution and the EMP position

Animal hemispheres dissected from unfertilized pigmented eggs that were cut in the equatorial plane and hybridized with *notch1* or *pou5f3.1* probes as described above were subjected to a morphometric analysis with FIJI/ImageJ as follows (Supplementary Figure S1). For determining the positions of the animal hemisphere’s and the ISH domain’s centroids, measurements were set to fit an ellipse. External views (animal view face images) of the egg’s animal hemisphere were used to obtain the animal hemisphere’s outline, the adjusted ellipse, and the centroid position through the Analyze Particles tool, as shown in Supplementary Figure S1. This centroid was named GAPc, as an estimate of the GAP position. Particles were added to the ROI Manager, and measurements gave us the GAPc coordinates, which were used to mark its position with the overlay brush. Then, the EMP was also marked with the overlay brush. The overlay containing the egg’s outline, the GAPc, and the EMP was flattened and saved with.png format. This image was then processed with Adobe Photoshop. After unlocking layers, an overlay with transparent background was generated by deleting everything (after selection with the magic wand or the lasso tool), except for the egg’s outline, the GAPc, and the EMP. To overlap this image to the ISH image (equatorial face) of the same egg, both were imported to CorelDRAW. The ISH image was digitally inverted to put it in register with the animal face view. In this way, all further measurements performed on the inverted ISH image are indeed projections on the animal view face, from which the egg’s outline, the GAPc, and the EMP positions were obtained. Then, the transparent overlay containing these landmarks was reoriented until the egg’s outline in the overlay matched the egg’s contour on the inverted ISH image. Both objects were then grouped and reoriented on a polar coordinates graph representing the animal view of the egg, with the *y*-axis corresponding to the egg’s meridian passing through the GAPc and the EMP, with the GAPc centered at x,y = 0, and the EMP in the positive ray of the *y*-axis. The *x*-axis corresponds to the meridian perpendicular to the GAPc-EMP meridian. We defined four quadrants around the GAPc, with boundaries at the *x* and *y*-axes. They were numbered clockwise, from q1 to q4 (Supplementary Figures S1–S4; Figure 5F). The CorelDRAW image generated in such a way was exported to.tif and cut with Adobe Photoshop to 15 cm × 15 cm at 300 ppi. This image was opened with FIJI/ImageJ and made binary to define a region of interest (ROI) encompassing the whole ISH domain (ISH ROI), while synchronizing the binary and the RGB image to trace the ISH ROI. After measuring to obtain the ISH ROI centroid, which we designated as ISH centroid (ISHc), this was marked in the overlay of the RGB image, together with the radius passing through the ISHc. A flattened image was created in tif format to proceed for further measurements, which were performed using the ObjectJ Plugin in FIJI/ImageJ, available at: https://sils.fnwi.uva.nl/bcb/objectj/. For this purpose, we created an ObjectJ project (Supplementary Methods) to measure the parameters listed in Table 1.

**TABLE 1:** Parameters for the morphometric analysis of ISH domains.

Parameter	Abbreviation	Definition
GAP centroid	GAPc	Centroid of the adjusted ellipse corresponding to the animal hemisphere outline. It represents a geometric animal pole (GAP) estimate
Egg’s maturation point	EMP	White spot left in the pigmented egg’s animal hemisphere after the extrusion of the first polar body during the transition to meiotic metaphase II.
*y*-axis length	YL	Measured length of the *y*-axis comprised by the egg’s outline (px)
Circle radius	CR	Calculated length (px): CR=0.5×YL
ISHc angle	ISHc Ө	Angle between the radius passing through the ISHc and the radius on the *y*-axis passing through the EMP, with the vertex in the GAPc
EMP-GAPc distance	EGD	Measured distance between the EMP and the GAPc (px)
ISHc-GAPc distance	IGD	Measured distance between the ISHc and the GAPc (px)
Relative EGD diametrical distance	rEGD_diam_ (%)	Calculated relative distance: expressed as % of YL. $rEGDdiam=\frac{EGD}{YL}\times 100$
Relative IGD diametrical distance	rIGD_diam_ (%)	Calculated relative distance: expressed as % of YL. $rIGDdiam=\frac{IGD}{YL}\times 100$
Relative EGD radial distance	rEGD_rad_ (%)	Calculated relative distance: expressed as % of CR. $rEGDrad=\frac{EGD}{YL}\times 100$
Relative IGD radial distance	rIGD_rad_ (%)	Calculated relative distance: expressed as % of CR. $rIGDrad=\frac{IGD}{YL}\times 100$
ISH area per quadrant	ISHa q_n_	Measured area of the ISH domain for each quadrant (q1 to q4) (px^2^)
Relative ISH area per quadrant	rISHa q_n_ (%)	Calculated relative area: expressed as % of total ISH area. $rISHa\;qn=\frac{ISHa\;qn}{ISHa\;q1+ISHa\;q2+ISHa\;q3+ISHa\;q4}\times 100$

The ISHc angle serves as an estimate of the position of the ISH domain in relation to the EMP. This angle may be in theory between 0° and 180°, with positive and negative values at the right and the left sides of the *y*-axis, respectively (Supplementary Figures S1–S4; Figure 5F). Those ISHc forming an angle between ± 89° and 0° were considered to be located on the same side of the EMP, whereas those forming an angle between ± 91° and 180° were considered to be located on the opposite side of the EMP.

Assuming a spheric egg shape, YL would represent the egg’s diameter if the egg were cut exactly at the equatorial plane. Then, one-half of this length would represent the egg’s radius (or circle radius, CR, at the equatorial plane). As it is impossible to ascertain if the eggs were cut exactly at the equatorial plane, to compensate for small deviations between samples, all measured distances were normalized by dividing them by the corresponding measured YL or calculated CR. For expressing results in the graphs, we used normalizations relative to the CR, thus representing relative radial distances, as they more clearly show the grade of eccentricity with respect to the GAPc.

It is obvious that the *y*-axis cannot be defined in eggs with a central EMP, i.e., those in which the EMP position coincides with the GAPc position. Moreover, we surmise that as the GAPc is an estimate of the GAP position and is subject to error, the orientation of the *y*-axis might be wrongly determined in eggs with an EMP very close to the GAPc. Therefore, a cut-off level for the distance between the EMP and the GAPc was assumed to minimize the error of orienting the egg along an ambiguous *y*-axis. Hence, for analyzing the spatial relationship between the EMP and the ISH domain, we excluded those eggs with a rEGD_rad_ < 10%, even if they showed an asymmetric *notch1* mRNA or a central *pou5f3.1* distribution when visually observed, although they are included in the qualitative analysis shown in Supplementary Table S3. We also excluded from this morphometric analysis those eggs included in Supplementary Table S3 in which the EMP was not visible because it was masked by the ISH staining.

For each frog and ISH marker, EMP and ISHc positions for the analyzed eggs were represented using CorelDRAW on a polar coordinates graph representing the animal view of an egg as a circle with a *y*-axis length and CR length of 100% on the equatorial plane, as described above (Supplementary Figures S1–S2; Figure 5; Supplementary Table S4). To construct the graphs in Supplementary Figures S2–S4, each point was positioned in the polar graphs using the relative radial distance to the GAPc (rEGD_rad_ and rIGD_rad_) and the ISHc angle (being the EMP positioned on the positive ray of the *y*-axis, by definition) (Supplementary Figure S1B). Then, the three polar graphs of Supplementary Figures S2–S4 were overlapped to construct the polar graph in Figure 5F containing the ISH centroids from all the analyzed eggs and both ISH markers. In this figure, to avoid overcrowding the graph with dots and to clearly show the position of the ISHc, we deleted the individual EMPs and instead represented the mean EMP position for all the analyzed eggs. This is a *bonafide* simplification, as there were no significant differences between the mean rEGD_rad_ of eggs analyzed for *notch1* and *pou5f3.1* nor between the mean rEGD_rad_ of eggs from the two frogs analyzed (two-tailed *t*-test, *p* < 0.05).

Statistical analysis was performed with GraphPad Prism. The employed tests are indicated in the figure legends or the main text. The difference between means was considered statistically significant when *p* < 0.05.

## Results

### Notch1 is asymmetrically distributed in *Xenopus laevis* zygotes before the onset of cortical rotation

**FIGURE 2:**
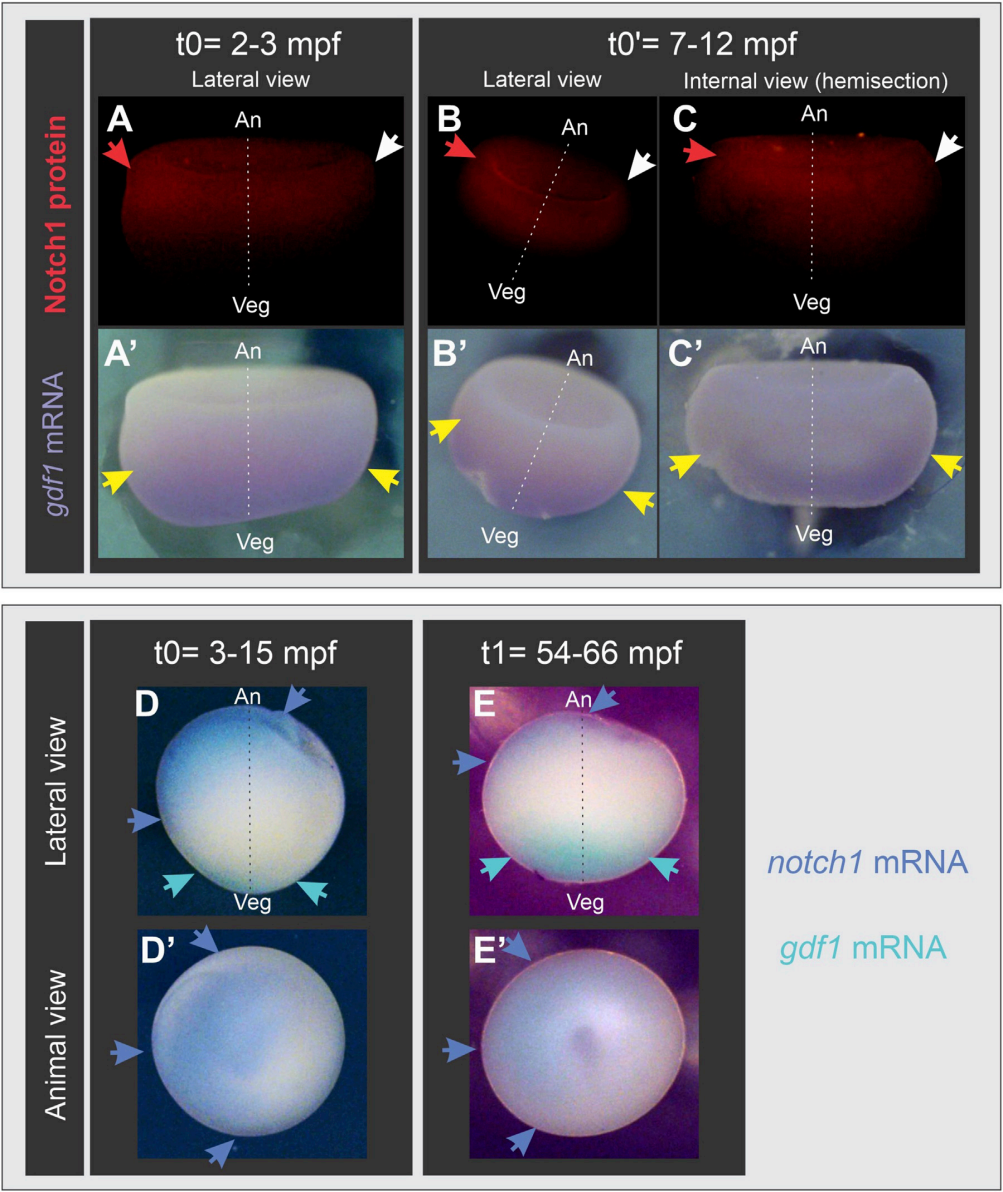
Notch1 protein and mRNA are asymmetrically distributed in the animal hemisphere of *Xenopus laevis* zygotes before the onset of cortical rotation. Albino embryos at s1 fixed before (t0, t0′) or at the end (t1) of cortical rotation; mpf, minutes post-fertilization. **(A’–E’)** are the same embryos shown in **(A–E)**, respectively. **(A–C’)** combined Notch1 immunofluorescence **(A–C)** and *gdf1 (vg1)* ISH **(A’–C’)**. Red and white arrows respectively point to the highest and lowest immunofluorescence signal, indicating that Notch1 protein is asymmetrically distributed in the animal hemisphere before and after cortical rotation, while *gdf1 (vg1)* mRNA is uniformly distributed in the vegetal region (yellow arrows), as expected. **(D–E’)** double ISH for *notch1* (blue) and *gdf1 (vg1)* (turquoise). Blue arrows point to the asymmetric distribution of *notch1* mRNA in the animal hemisphere before **(D,D’)** and at the end of cortical rotation **(E,E’)**. Embryo’s orientation was verified by *gdf1* (*vg1*) mRNA location (turquoise arrows) as reference since it is uniformly distributed in the vegetal cortex. An, animal pole; Veg, vegetal pole; broken line, animal-vegetal axis.

From our previous findings and given the well-known relocation of maternal dorsal determinants through cortical rotation, we wondered if the early asymmetric location of Notch1 precedes or appears during cortical rotation. For this purpose, we analyzed the distribution of Notch1 protein and mRNA through immunofluorescence and *in situ* hybridization (ISH) in albino embryos at different post-fertilization timepoints, before the onset of cortical rotation, which begins around 30 min post-fertilization (mpf), and at the end of this process. Because the animal-vegetal axis cannot be assigned in albino embryos by means of pigment distribution, we revealed *gdf1* mRNA in the same embryos, as this marker (previously known as *vg1*) is uniformly located in the vegetal hemisphere of oocytes and eggs, and is inherited by the vegetal cells of early embryos without being translocated to the dorsal side (Weeks and Melton, 1987; Alarcón and Elinson, 2001). Surprisingly, we found that both Notch1 protein (Figures 2A–C) and mRNA (Figure 2D) are asymmetrically distributed in the animal hemisphere before cortical rotation takes place (Supplementary Table S1), even as early as at 3 mpf (Figure 2A). This *notch1* mRNA asymmetry was maintained by the end of cortical rotation (Figure 2E). Our results demonstrate that an early asymmetric Notch1 distribution exists in the zygote’s animal hemisphere, and it is independent of cortical rotation.

### Notch1 protein is asymmetrically distributed before fertilization in an axis orthogonal to the animal-vegetal axis of *Xenopus laevis* eggs

The asymmetric distribution of *notch1* gene products in the animal hemisphere of zygotes preceding cortical rotation might be due to an inherited maternal asymmetry. Therefore, we addressed if such asymmetries could be present prior to fertilization in eggs.

**FIGURE 3:**
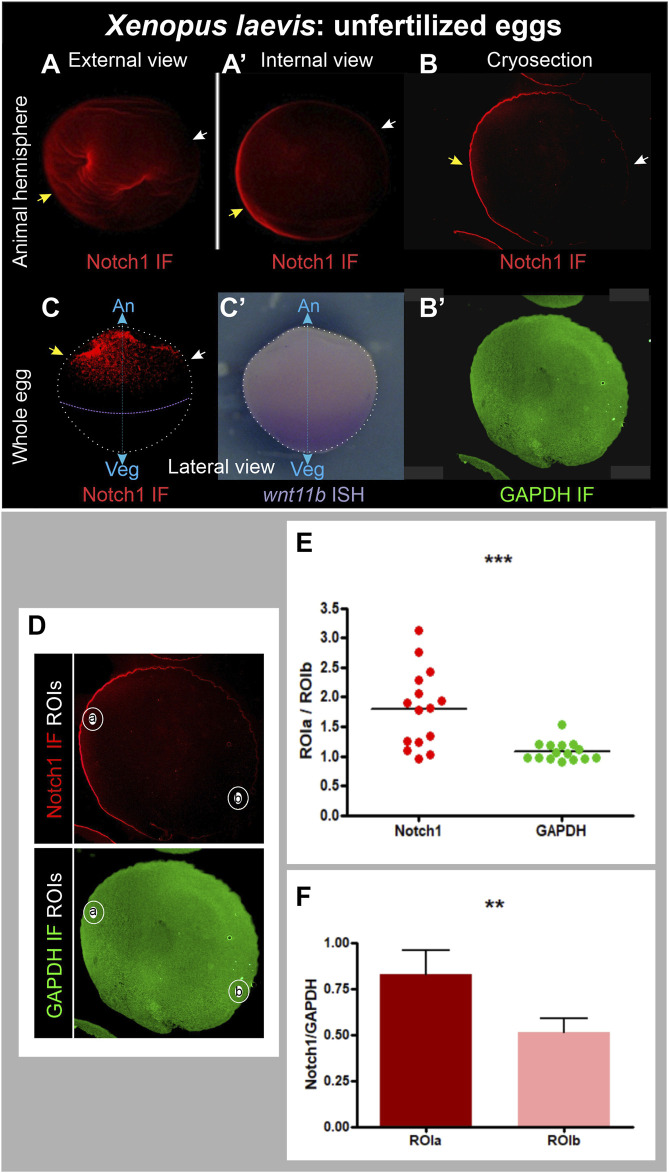
Asymmetric distribution of Notch1 protein in the animal hemisphere of unfertilized *Xenopus laevis* eggs. **(A–B’)** Pigmented animal hemispheres were dissected by cutting them away through the equatorial plane, bleached, and directly processed for Notch1 immunofluorescence **(A,A’)** or cryosections followed by Notch1 immunofluorescence combined with GAPDH immunofluorescence as ubiquitous reference protein **(B,B’)**. **(C,C’)** Whole albino egg, Notch1 immunofluorescence **(C’)** combined with *wnt11b* ISH as reference to verify the orientation of the animal-vegetal axis **(C’)**, since *wnt11b* mRNA is uniformly distributed in the vegetal region. Yellow and white arrows respectively point to the highest and lowest Notch1 immunofluorescence signal, indicating that Notch1 protein is asymmetrically distributed in the animal hemisphere in unfertilized eggs. An, animal pole; Veg, vegetal pole; IF, immunofluorescence; cyan double arrow, animal-vegetal axis. **(D–F)** Quantification of Notch1 and GAPDH immunofluorescence in cryosections of animal hemispheres obtained from pigmented, unfertilized eggs, bleached before immunofluorescence. The images shown in **(D)** are the same as in **(B,B’)**, but here, the selected ROIs are demarcated. **(E)** Dispersion graph comparing the relative mean pixel intensity (mpi) between ROIa and ROIb for Notch1 and GAPDH immunofluorescence in animal hemisphere cryosections from 15 independent unfertilized eggs. The mean ROIa/ROIb ratio was significantly higher for Notch1 than for GAPDH immunofluorescence, which was close to 1, as expected for a protein of homogeneous distribution (*p* = 0.0008, two-tailed paired *t*-test). **(F)** Comparison of relative Notch1/GAPDH mpi levels between ROIa and ROIb in the same set of eggs shown in **(E)**. The Notch1/GAPDH ratio was significantly higher in ROIa than in ROIb (*p* = 0.0016, two-tailed paired *t*-test, n = 15). Since each sample always comprised a pair of measurements (as defined by ROIa and ROIb), paired t-tests (two-tailed) were performed; the difference between means was considered statistically significant when *p* < 0.05.

We dissected animal hemispheres (where Notch1 protein and mRNA are found during the early embryonic stages) from fixed, unfertilized *X. laevis* eggs obtained from pigmented females and processed them for immunofluorescence. Notch1 asymmetries in the animal hemisphere were observed in 96 out of 99 eggs obtained from three different pigmented females (Supplementary Table S2; Figure 3A,A′-C; Supplementary Figure S5A–C). We also fixed unfertilized eggs from albino females and combined Notch1 immunofluorescence with *wnt11b* ISH to orient the unpigmented eggs in the animal-vegetal axis since *wnt11b* transcripts are localized in a gradient from the vegetal pole to the equator before fertilization (Kataoka et al., 2005). Notch1 was asymmetrically distributed in the animal hemisphere of eggs from two different albino females (Figure 3C,C′; Supplementary Table S2).

A potential problem with immunofluorescence in egg halves or whole eggs might be that deformations could arise, resulting in an apparent asymmetric signal due to the unequal accumulation of material. Therefore, we performed double immunofluorescence for Notch1 and the ubiquitously expressed proteins GAPDH or α-Tubulin in cryosections obtained from pigmented hemispheres in a plane perpendicular to the animal-vegetal axis. In this way, we could observe the same density of material throughout the entire sample. Again, we found a very clear asymmetric distribution of Notch1 immunofluorescence (Figure 3B), while GAPDH (Figure 3B′) or α-Tubulin immunofluorescence (Supplementary Figure S5D, E) in the same sections was ubiquitous, as expected. These asymmetries in cryosections were observed in 21 out of 22 eggs obtained from two different females (Supplementary Table S2), confirming that Notch1 protein is asymmetrically distributed in the animal hemisphere of *X. laevis* unfertilized eggs in an axis orthogonal to the animal-vegetal axis. Overall, this asymmetric Notch1 protein distribution was observed in 96% of the eggs analyzed by either experimental approach (n = 130; 6 independent biological replicates; Supplementary Table S2).

For further verification, Notch1 and GAPDH immunofluorescence were quantified in cryosections of animal hemispheres from 15 independent unfertilized eggs by measuring the mean pixel intensity (mpi) in two circular regions of interest (ROI), one in the region of visually maximum Notch1 immunofluorescence (ROIa) and the other, in the opposite side (ROIb) (Figure 3D). Figure 3E shows a dispersion graphic comparing the ROIa/ROIb mpi ratios between Notch1 and GAPDH. For GAPDH immunofluorescence, these ratios were consistently distributed around a mean close to 1 (mean = 1.079 ± 0.04231, n = 15), as expected for a protein of homogeneous distribution. In contrast, for Notch1 immunofluorescence, ROIa/ROIb mpi ratios were distributed around a significantly higher mean (1.801 ± 0.1699, n = 15; *p* = 0.0008, two-tailed paired *t*-test) (Figure 3E), confirming that Notch1 protein is asymmetrically distributed in the animal hemisphere, whereas GAPDH is homogeneously distributed, as expected. Moreover, when we normalized Notch1 in relation to GAPDH immunofluorescence levels, the Notch1/GAPDH ratio for ROIa was significantly higher than the Notch1/GAPDH ratio for ROIb (*p* = 0.0016, two-tailed paired *t*-test, n = 15) (Figure 3F). In conclusion, Notch1 protein is asymmetrically distributed in the animal hemisphere of *X. laevis* unfertilized eggs.

### *notch1* mRNA is asymmetrically distributed in the animal hemisphere of unfertilized *Xenopus laevis* eggs and it is not the only maternal transcript with asymmetric distribution

Next, we wondered if *notch1* transcripts and other mRNAs encoded by genes involved in Notch signaling (*dll1, hes4*) (Turner and Weintraub, 1994; Chitnis et al., 1995; Davis et al., 2001; Tsuji et al., 2003; López et al., 2005; Sakano et al., 2010), embryonic dorsoventral patterning (*bmp4*) (Hemmati-Brivanlou and Thomsen, 1995; Bell et al., 2003; Reversade et al., 2005; Kirmizitas et al., 2017)*,* or maintenance of pluripotency (*pou5f3.1*) (Morrison and Brickman, 2006) showed asymmetric patterns in unfertilized eggs in an axis different from the animal-vegetal one. Single ISH for each marker was performed in dissected animal hemispheres from pigmented eggs, or double ISH for *notch1* and *wnt11b* as reference marker for the vegetal pole was performed in albino eggs. We also performed double ISH for *notch1* and *pou5f3.1* in albino eggs.

**FIGURE 4:**
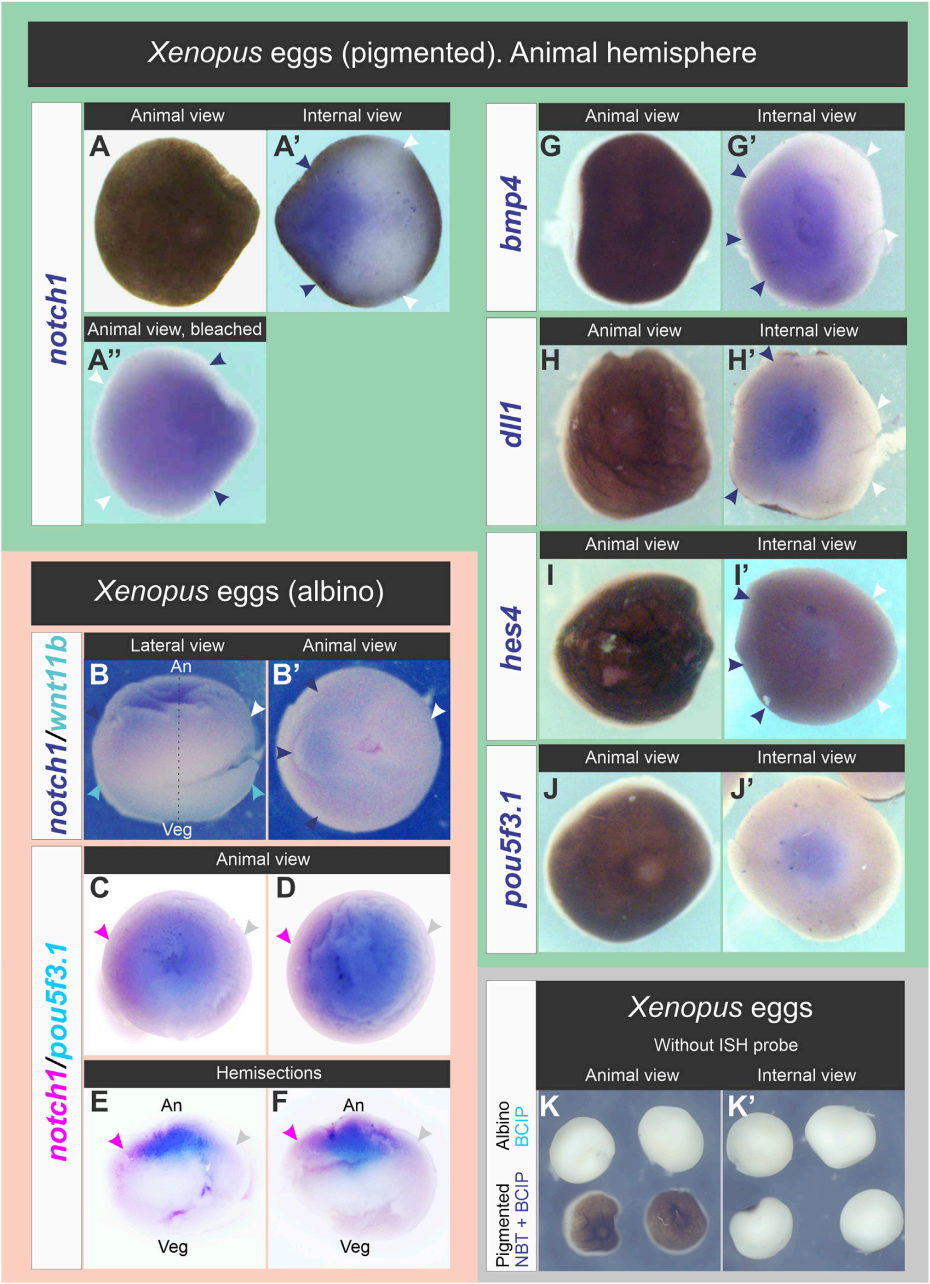
Distribution of *notch1* mRNA and other transcripts in the animal hemisphere of unfertilized *Xenopus laevis* eggs. **(A–A”)**, **(G–J′)** Pigmented eggs were cut through the equatorial plane. Animal hemispheres were processed for ISH for the following markers: *notch1* **(A–A”)**, *bmp4* **(G,G′)**, *dll1* **(H,H′)**, *hes4* **(I,I′)**, and *pou5f3.1* **(J,J′)**. Animal hemispheres in **(A,G–J)** were photographed in animal view, then turned 180° to the right of the observer to photograph their internal side (equatorial face of the animal hemisphere; internal views in **(A′,G′-J′)**. The animal hemisphere processed for *notch1* ISH was photographed before **(A,A′)** and after bleaching **(A**″**)** in animal **(A,A**′**)** and internal views **(A′)**. **(B,B′)** Albino egg processed for double ISH for *notch1* (blue) and *wnt11b* (turquoise) shown in lateral **(B)** and animal views **(B′)**. Dark blue and white arrowheads respectively point to the highest and lowest *notch1* ISH signal, indicating that its transcripts are asymmetrically distributed in the animal hemisphere. The egg’s orientation was verified by the location of *wnt11b* mRNA as reference (turquoise arrowheads), which is uniformly distributed in the vegetal cortex **(B)**. **(C–F)** Whole albino eggs processed for double ISH for *notch1* (magenta) and *pou5f3.1* (turquoise). Animal **(C,D)** and internal views of hemisections cut in the animal-vegetal plane **(E,F)**. Magenta and gray arrowheads respectively point to the highest and lowest *notch1* ISH signal, indicating that its transcripts are enriched on one side of the animal hemisphere, while *pou5f3.1* does not show such asymmetry. An, animal pole; Veg, vegetal pole; broken line, animal-vegetal axis. Dark blue and white arrowheads in **(A′-A**″**,G′–I′)** respectively point to the highest and lowest levels of *notch1, bmp4, dll1*, and *hes4* transcripts in the animal hemisphere. Notice the central location of *pou5f3.1* transcripts in the animal hemisphere, most likely related to accumulation in the nuclear region. **(K,K′)** Absence of non-specific staining in whole albino eggs (upper row) and animal hemispheres from pigmented eggs (lower row) that were processed for ISH but without adding probes, in parallel with albino eggs processed for double ISH (as those shown in B,B′) and revealed with BCIP and with eggs processed for *notch1* ISH (as shown in A-A″) and revealed with NBT + BCIP. After the ISH procedure, albino eggs were bisected to show the internal face (K′, upper row).

*notch1* mRNA was asymmetrically distributed in the animal hemispheres (Figure 4A–A″,B-F; 97%, n = 70 unfertilized eggs obtained from a total of seven different females; Supplementary Table S3). Surprisingly, the same was observed for *bmp4* (Figure 4G,G′), *dll1* (Figure 4H,H′), and, although weakly, also with *hes4* transcripts (Figure 4I,I′) (Supplementary Table S3). In contrast, we observed a central, non-asymmetric distribution of *pouf5f3.1* mRNA (Figures 4C–F, J,J′; Supplementary Table S3), probably representing accumulation in the egg’s nuclear region below the animal pole. Eggs processed in parallel without adding the ISH probe did not show any staining (Figure 4K,K′), confirming that the patterns shown here were not due to non-specific color reaction. These results demonstrate that in unfertilized eggs, there is a prepattern of asymmetric distribution of some maternal mRNAs, some of them with known ventralizing properties, such as *notch1* and *bmp4*, in an axis different from the animal-vegetal axis. Asymmetric distribution of *bmp4* transcripts in the egg’s animal hemisphere as shown in the present work was not previously recognized, even when the ISH image in Figure 2A in Bell et al. (Bell et al., 2003) suggests being so.

### *notch1* mRNA preferentially locates on the EMP side in *Xenopus laevis* unfertilized eggs

**FIGURE 5:**
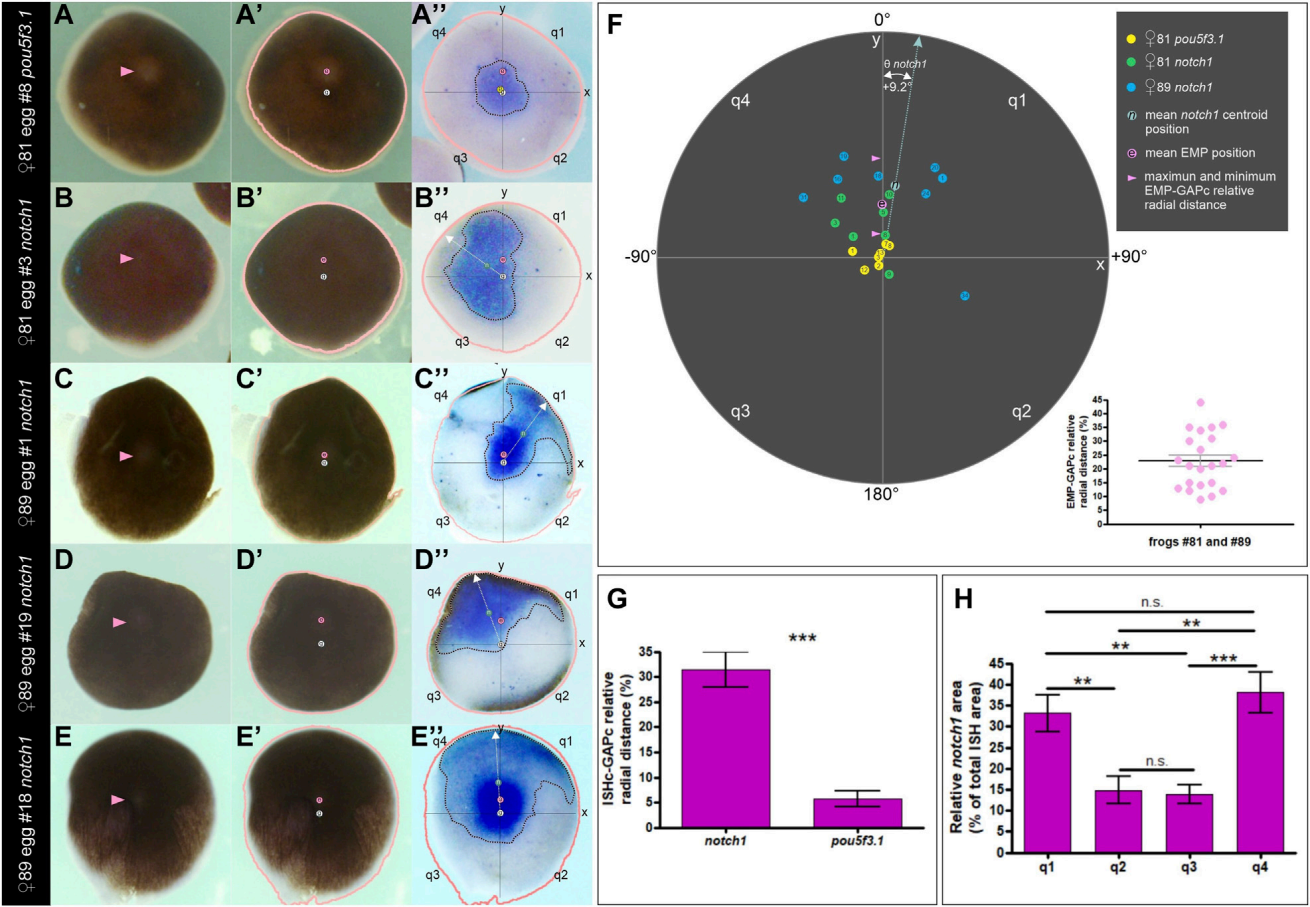
Morphometric analysis showing that the distribution of *notch1* mRNA is biased towards the EMP side in unfertilized *Xenopus laevis* eggs. **(A–E**″**)** Examples of animal hemispheres obtained from pigmented eggs that were analyzed by morphometry. **(A–A**″**)** *pou5f3.1* ISH. **(B–E**″**)** *notch1* ISH in four different animal hemispheres. The first column shows the external, animal views; pink arrowheads point to the EMP. The second column shows the same animal views labeled with the EMP (e, pink circles) and GAPc (g, white circles) locations, and a salmon line corresponding to the egg’s outline drawn by the Analize Particle tools with FIJI. In the third column, these landmarks were overlapped as an overlay to the inverted internal views (equatorial face) showing the ISH domains of the corresponding eggs. Axes and quadrants were labeled as described in the text. Dotted black lines demarcate the ISH ROIs; their centroids are labeled with a yellow circle for *pou5f3.1* (p) and a green circle for *notch1* (n). The white dotted arrow lines correspond to the radius containing the ISH centroids (ISHc). Eggs in **(A–A**″**,D–D**″**)** are the same as Figure 4F, F′ and A-A″, respectively, but here, their images are oriented for the morphometric analysis and labeled with the landmarks. **(F)** Polar coordinates graph showing the distribution of ISH centroids for *pou5f3.1* and *notch1* (color-coded circles, see references in the upper right corner of the figure) for all the analyzed eggs, according to their relative radial distances to the GAPc and their deviation angle from the EMP-GAPc axis (*y*-axis). Inside the circles, numbers indicate the individual egg# (see Supplementary Table S4 for the corresponding measurements). For *notch1* ISH centroids, the mean deviation angle Ө from the EMP-GAPc axis is indicated. The pink circle shows the mean EMP relative radial distance to the GAPc for all analyzed eggs; pink arrowheads show the range of EMP relative positions of the analyzed eggs. Notice that the inferior range limit is the cut-off level assumed for performing the morphometric analysis. The inset in the lower right corner shows a dispersion diagram representing the EMP relative radial distance to the GAPc for all the analyzed eggs. Mean±SEM values are indicated (black and grey lines, respectively). Their spatial distribution is displayed in the polar coordinates graphs of Supplementary Figures S2–S4 and measurements are shown in Supplementary Table S4. See Supplementary Movie S1 showing the transitions between images. **(G)** ISH centroids for *notch1* mRNA are significantly eccentric in relation to the central marker *pou5f3.1* (unpaired, two-tailed *t*-test; *p* < 0.0001). Bars indicate mean±SEM. **(H)** Spatial composition of the *notch1* ISH domain. Both quadrants on the same side of the EMP (q1, q4) show a significantly higher contribution to the ISH area than the opposite quadrants (q2, q3) in the analyzed eggs (two-tailed, paired *t*-test; q1,q2: *p* = 0.0052; q1,q3: *p* = 0.0083; q1,q4: *p* = 0.5519; q2,q3: *p* = 0.7671; q2,q4: *p* = 0.0089; q3,q4: *p* = 0.0003); n.s., non-significant differences. Bars indicate mean±SEM. *notch1* ISH, n = 15, two independent females; *pou5f3.1* ISH, n = 7, one female.

Next, we aimed to determine if the asymmetric distribution of *notch1* mRNA bears a spatial correlation with the EMP location since it was previously established that this point is generally eccentric in relation to the GAP and predicts the direction of yolk mass rotation in activated eggs (Brown et al., 1994). For this purpose, we performed a morphometric analysis of the ISH domains in pigmented animal hemispheres from unfertilized eggs, comparing *notch1* and *pou5f3.1* mRNA distributions as detailed in the Materials and Methods section (see Table 1; Supplementary Figure S1). The animal hemisphere centroid was considered as an estimate of the GAP and was named GAP centroid (GAPc). We represented the egg’s animal hemisphere in animal view as a circle in a polar coordinates graph with the EMP-GAPc axis as the *y*-axis, the GAPc in the center (at the origin of coordinates x,y), and projected the ISH domain into this template. Then, we measured the relative radial distance of the ISH domains centroids (ISHc) to the GAPc and their angular deviation from the EMP-GAPc axis (*y*-axis). We also measured the total ISH area and the relative ISH area contributed by each of the four quadrants defined around the GAPc (q1 to q4, in clockwise direction). Results are presented in Figure 5, Supplementary Figures S2–S4, Supplementary Movie S1; Supplementary Table S4.

The *pou5f3.1* centroids were found very closely around the GAPc (mean relative radial distance = 5.7%, n = 7; Figure 5A-A″,F,G; Supplementary Figure S2, Supplementary Movie S1), consistent with a central location for this marker. In contrast, the distribution of *notch1* centroids clearly did not overlap that of the *pou5f3.1* centroids (Figures 5B–F; Supplementary Figure S3; Supplementary Figure S4, Supplementary Movie S1). Moreover, they were significantly farther away from the GAPc (mean relative radial distance = 31.5%, n = 15, *p* < 0.05; two-tailed *t*-test; Figure 5G), implying that they are consistently eccentric (i.e., 5.5 times more eccentric than the central marker *pou5f3.1*), hence reflecting *notch1* mRNA asymmetric distribution in the animal hemisphere. Notably, 13 out of the 15 analyzed eggs showed the *notch1* centroid on the same side of the EMP (Figure 5F; Supplementary Figures S3, S4; Supplementary Table S4), being distributed at both sides of the EMP-GAPc axis. Overall, the mean deviation angle of the *notch1* ISHc from the EMP-GAPc axis was +9.2° (Figure 5F, Supplementary Movie S1; Supplementary Table S4). Morphometric analysis of the *notch1* ISH area showed that both quadrants on the same side of the EMP (q1 and q4) mostly contribute to the ISH domain in the analyzed population of eggs in equal proportions, with significantly lower contribution from both quadrants on the opposite side of the EMP (q2 and q3) (paired, two-tailed *t*-test, *p* < 0.05; n = 15) (Figure 5H; Supplementary Table S4). These results confirm the asymmetric distribution of *notch1* transcripts in the animal hemisphere and strongly argue in favor of a bias of *notch1* mRNA distribution towards the EMP side in unfertilized eggs.

### Notch1 is asymmetrically distributed in *Xenopus laevis* oocytes

The results shown above prompt the question about the distribution of Notch1 during oogenesis. In the ovary of adult *X. laevis* females, oocytes are found in meiotic prophase I at different growing stages classified from I to VI (Dumont, 1972). Stage VI oocytes (sVI) represent their terminal stage. They are competent for maturation (during which resumption of meiosis from prophase I to metaphase II occurs) and subsequent ovulation, which proceed under the stimulus of pituitary and ovarian hormones (Rasar and Hammes, 2006).

To address when Notch1 expression and asymmetry in the animal hemisphere first arise, defolliculated sI to sVI oocytes obtained from pigmented females were processed for *wnt11b* ISH, as reference marker of the vegetal pole throughout oogenesis (Kloc and Etkin, 1995), followed by Notch1 immunofluorescence. After the ISH staining, a bleaching step was included to avoid quenching of the immunofluorescence by the pigment.

**FIGURE 6:**
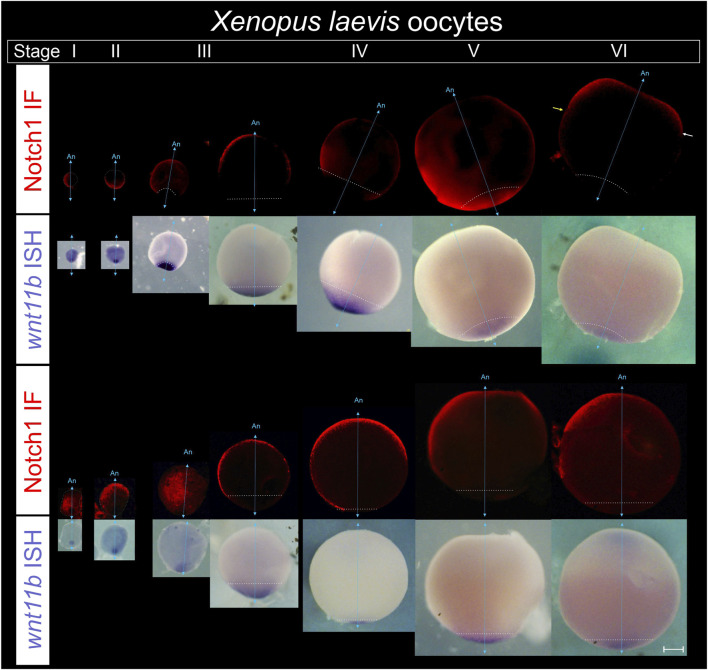
Notch1 protein becomes asymmetrically distributed in an orthogonal axis with respect to the animal-vegetal axis during *Xenopus laevis* oogenesis. Defolliculated oocytes obtained from pigmented females were fixed, bleached, and processed for combined immunofluorescence for Notch1 protein and ISH for *wnt11b* mRNA as reference marker of the vegetal pole to orient the oocytes in their animal-vegetal axis. From left to right, successive stages of oogenesis are shown in two representative series. Oocytes were classified according to the stages (s) described by (Dumont, 1972). For each series, the upper tier shows Notch1 immunofluorescence. The same oocytes are shown in bright field in the lower tier, with *wnt11b* mRNA located in the vegetal pole throughout oogenesis and also on the cytoplasm of early sI oocytes, as previously described (Kloc and Etkin, 1995). An, animal pole; cyan double arrow, animal-vegetal axis, as determined by *wnt11b* mRNA location; the white dotted line demarcates the contour of sI and sII oocytes and the *wnt11b* domain from sIII to sVI. Yellow and white arrows on the sVI oocyte point to the vegetal boundary of Notch1 protein expression in the animal hemisphere, which is more vegetal on one side (yellow arrow) than on the other side of the picture (white arrow), thus showing an asymmetric domain in the animal hemisphere. Scale bar: 200 µm. See data in Supplementary Table S5.

We found that Notch1 is asymmetrically distributed towards one side of the animal-vegetal axis from the earliest stages of oogenesis (Figure 6; Supplementary Table S5). We often observed Notch1 immunofluorescence in the vegetal region with variable intensity during earlier stages, but expression appears to be more restricted towards one side of the animal hemisphere by stage VI (Figure 6). Therefore, Notch1 asymmetry in an axis orthogonal to the animal-vegetal axis emerges with oogenesis.

### *Notch1* mRNA is asymmetrically distributed in zebrafish during early embryogenesis

**FIGURE 7:**
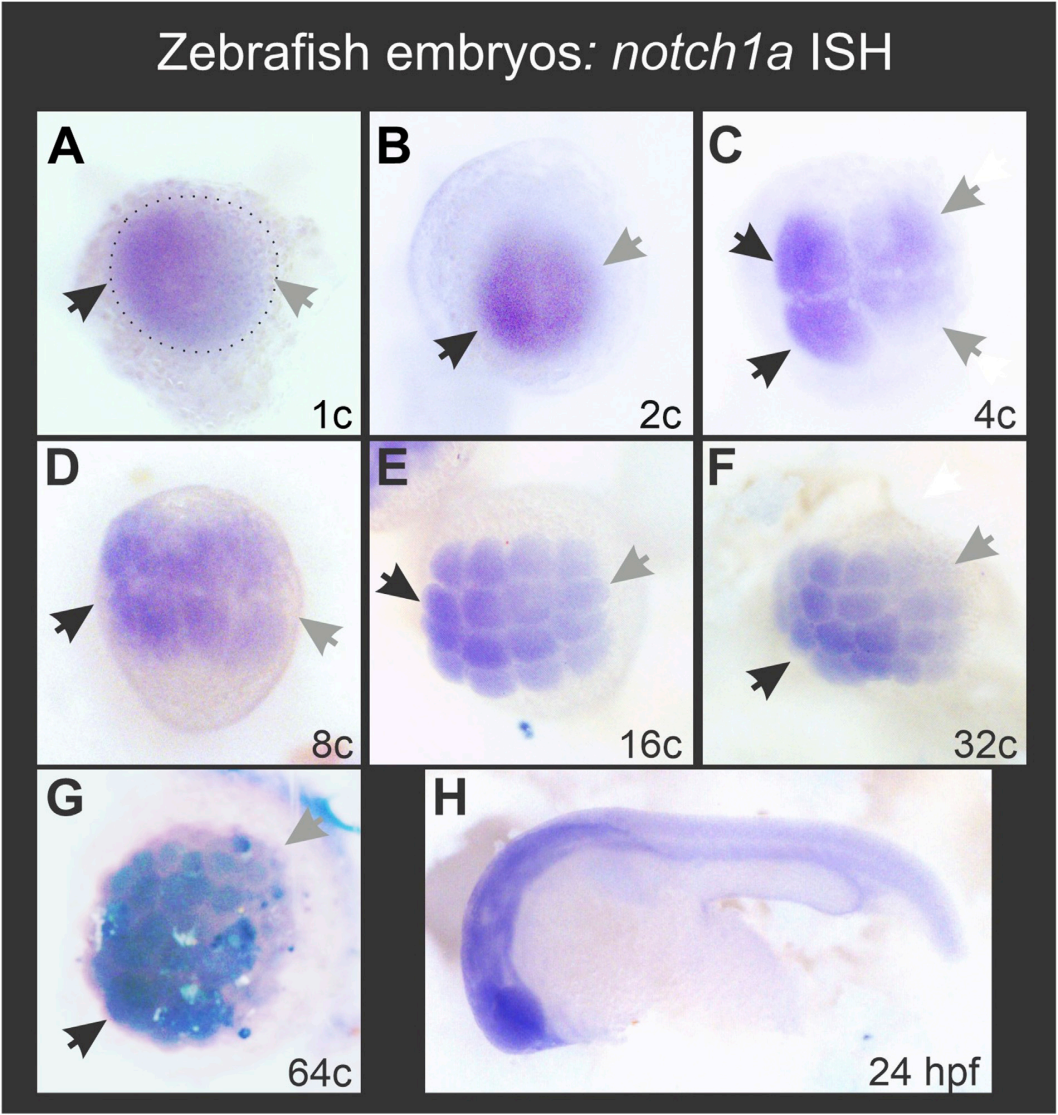
Zebrafish *notch1a* mRNA is asymmetrically distributed during early embryogenesis in the animal hemisphere from the 1-cell stage. Black and grey arrows in **(A–G)** respectively point to the regions with the highest and lowest *notch1a* ISH signal. The dotted ellipse in **(A)** demarcates the blastodisc. **(H)** ISH performed in parallel with the same *notch1a* probe in a 24 hpf embryo, showing typical expression for this transcript.

We have previously shown that *notch1* transcripts are enriched on the ventral side during early *X. laevis* embryogenesis (Castro Colabianchi et al., 2018) and we wondered if the same occurs in another anamniote model, like the zebrafish, in which we also showed an asymmetric Notch1 distribution at the protein level (Castro Colabianchi et al., 2018). *D. rerio* has four *notch* genes: *notch1a, notch1b, notch2* and *notch3*. The two *notch1* genes appeared in teleosts at a recent duplication event, after they diverged from tetrapods. Among them, *notch1a* transcripts are the only ones that can be detected by ISH as maternal mRNA (Favarolo and López, 2018), but no attention has been paid to whether it shows an asymmetric distribution. Therefore, we analyzed the expression of *notch1a* by ISH in zebrafish embryos and found that there is also a consistent enrichment of *notch1* transcripts in a region of the animal hemisphere, from the zygote stage until the last analyzed stage (sphere) (100% of embryos, n = 134; Figure 7; Supplementary Table S6). This result indicates that the early asymmetry in the distribution of *notch1* transcripts is conserved between fish and amphibians.

## Discussion

### Molecular asymmetries support the hypothesis of a latent dorsoventral prepattern in unfertilized *Xenopus laevis* eggs

In the present work, we found that the asymmetric distribution of Notch1 protein in an axis orthogonal to the animal-vegetal axis is already present from the earliest stages of oogenesis. This asymmetry is maintained and even more pronounced in mature, unfertilized eggs, in which a marked enrichment of Notch1 protein is observed towards one side of the animal hemisphere. Strikingly, in mature, unfertilized eggs, *notch1* mRNA is consistently enriched on the EMP side. After fertilization, *notch1* mRNA and its encoded protein are asymmetrically distributed in the zygote’s animal hemisphere before and after cortical rotation, and we have previously demonstrated that ventrally accumulated Notch1 normally plays a ventralizing role in the establishment of the embryonic dorsoventral axis (Acosta et al., 2011; Castro Colabianchi et al., 2018). Importantly, both phosphorothioate-modified antisense DNA oligonucleotides and antisense morpholino oligonucleotides have been previously used to block *notch1* function in oocytes by other authors (Mir et al., 2008) and early embryos in our previous work (Acosta et al., 2011; Castro Colabianchi et al., 2018). Since maternal mRNAs can be efficiently depleted from oocytes (but not from fertilized eggs) using phosphorothioate-modified antisense DNA oligonucleotides that trigger specific RNAse-H-mediated degradation, this technique is useful to assess maternal but not zygotic functions, whereas antisense morpholino oligonucleotides that block translation impair both maternal and zygotic functions (Hulstrand et al., 2010). The antisense *notch1* phosphorothioate-modified oligonucleotide efficiently depleted the endogenous *notch1* mRNA from oocytes and when they were transferred to host females, they could be fertilized. Endogenous *notch1* mRNA remained depleted in the resulting embryos through mid-blastula and at least until early gastrula. Noteworthy, when analyzed at the blastula stage, these embryos showed a remarkable expansion of the dorsal marker *foxi1* towards the ventral side (Mir et al., 2008), much similar to the ventral expansion of *bonafide* markers of the dorsal center that we observed after blocking *notch1* translation by means of morpholino injections in early embryos (Acosta et al., 2011), which also suppressed *bonafide* markers of the ventral center (Castro Colabianchi et al., 2018). Therefore, both depletion of *notch1* mRNA specifically from oocytes and inhibition of *notch1* translation in early embryos produced dorsalized phenotypes. We surmise that if *notch1* mRNA is destroyed before fertilization, there will not be a post-fertilization *notch1* mRNA asymmetry, because there simply will be no *notch1* mRNA to construct such asymmetry at the zygote stage. All this evidence indicates that sustained maternal *notch1* mRNA asymmetry, already established before fertilization, is necessary for the correct establishment of the initial dorsoventral patterning.

We have previously performed *notch1* gain-of-function and knock-down experiments in *X. laevis* zygotes and obtained a range of ventralized and dorso-anteriorized phenotypes, respectively, at tailbud stages (Acosta et al., 2011). These results were consistent with the changes we observed in the expression of dorsal and ventral markers at late blastula (Acosta et al., 2011; Castro Colabianchi et al., 2018). In the future, it will be interesting to perform *notch1* overexpression and knock-down experiments with phosphorothioate-modified antisense DNA oligonucleotides and antisense morpholinos in oocytes, *in vitro* mature them, and perform the host-transfer technique to obtain embryos (Mir and Heasman, 2008; Schneider et al., 2010). This will allow us to compare the phenotypes in tailbud stages with those previously obtained. A higher severity or penetrance would further support the maternal contribution of *notch1* to dorsoventral patterning.

The conclusion that Notch1 mRNA and protein are asymmetrically distributed along an axis orthogonal to the animal-vegetal axis before fertilization and cortical rotation are based on the uneven distribution of the immunofluorescence signal and colorimetric staining along the animal hemispheres. One potential caveat about this interpretation is that this uneven distribution might be due to technical issues. Since there is no marker currently available that could label the prospective ventral or dorsal sides at these stages to validate our results, several approaches were employed to gain robustness and consistency. First, we have analyzed large sample sizes with independent biological replicates and performed statistical analyses whenever possible. Then, we have also used double staining with known vegetal markers to confidently orient the animal-vegetal axis or with double immunofluorescence for the ubiquitously expressed proteins GAPDH or α-Tubulin in pigmented animal hemispheres, both in whole-mount and in cryosections to rule-out the possibility of observing an uneven distribution of Notch1 due to the unequal accumulation of material across the specimens.

In a recent RNA seq study along the animal-vegetal axis in *X. laevis* eggs, *notch1* transcripts were found within the animal group of maternal mRNAs (Supplementary Dataset S1 in Sindelka et al.) (Sindelka et al., 2018). However, the RNAseq study did not address the possibility of uneven distribution of transcripts in an axis orthogonal to the animal-vegetal one. While the mechanisms that control mRNA location in the vegetal pole of *Xenopus* oocytes and eggs were studied quite deeply and involve motifs in their 3′UTR sequences, those controlling animal mRNA locations are largely unknown (Sindelka et al., 2018).

In addition to *notch1*, *bmp4, dll1, and hes4* transcripts (although weakly for the latter) also showed a bias towards one side of the animal hemisphere in unfertilized eggs. Therefore, mRNAs corresponding to two pathways involved in ventral polarization are already asymmetrically distributed in an axis orthogonal to the animal-vegetal one before fertilization. They might be the source for localized translation during early embryogenesis before the beginning of zygotic transcription. So far, asymmetries of RNA or maternal proteins distribution have not been previously shown in *Xenopus* unfertilized eggs in an axis other than the animal-vegetal one.

**FIGURE 8:**
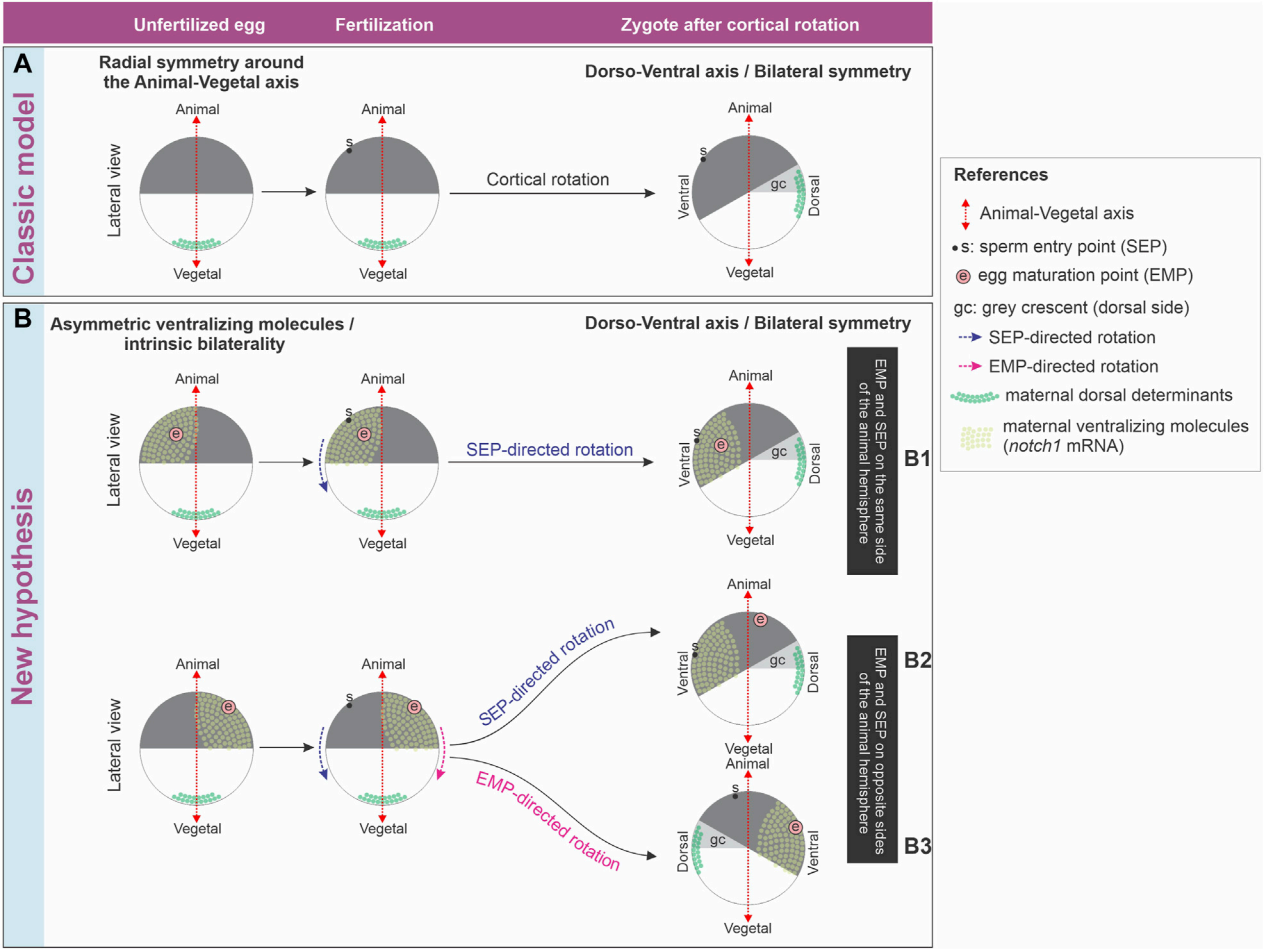
**(A)** Classic model of axis formation in *Xenopus*, which only considers relocation of maternal dorsal determinants after fertilization. **(B)** New hypothesis for axis formation in *Xenopus*, which considers both relocation of maternal dorsal determinants after fertilization and an intrinsic bilaterality of unfertilized eggs harboring an asymmetric distribution of ventralizing molecules in the animal hemisphere. See main text for additional details.

The EMP/*notch1* side likely predicts the provisional ventral side in unfertilized eggs, because 1) in unfertilized, activated eggs with an eccentric EMP, the yolk mass rotates towards the EMP side whereas in fertilized eggs, the yolk mass rotates in most of them towards the SEP, which generally predicts the ventral side (Brown et al., 1994); 2) if they are provided with a diploid nucleus and a centrosome, activated eggs can parthenogenetically develop into an embryo with the position of the dorsal midline predicted by the direction of rotation (Black and Vincent, 1988; Gerhart et al., 1989; Ubbels, 1997; Tassan et al., 2017); 3) *notch1* mRNA and protein are ventrally enriched in 1-cell embryos through cleavage stages and at least, until mid-blastula transition (Castro Colabianchi et al., 2018); 4) *notch1* has ventralizing properties in embryos and normally contributes to the establishment of a well-balanced dorsoventral axis. It is necessary for the expression of ventral center genes and prevents the expression of dorsal genes in the ventral side (Mir et al., 2008; Acosta et al., 2011; Castro Colabianchi et al., 2018). If *notch1* mRNA marks the provisional ventral side before fertilization (Figure 8B), then, its distribution should be conserved when the sperm cues fail to direct cortical rotation and the EMP-dependent directionality is used as a backup mechanism (Figure 8B, row B3) or when the EMP and SEP happen to be on the same side of the animal hemisphere (Figure 8B, row B1), but should be reorganized when the sperm redirects the cortical rotation cues if the EMP happens to be on the opposite side of the SEP and cannot counteract the SEP driving force (Figure 8B, row B2).

In conclusion, the discussion of whether an intrinsic bilaterality of the egg exists and participates in the axialization of the body plan is still active. The classic model of axis formation only considers relocation of maternal dorsal determinants from the vegetal pole to the future dorsal side as a consequence of sperm-directed cortical rotation but does not take into account the influence of early localized ventralizing molecules (Figure 8A). The molecular asymmetries shown in this work support the hypothesis that *X. laevis* unfertilized eggs possess inherent bilaterality. We propose that a maternal prepattern involving the asymmetric distribution of ventralizing molecules like Notch1 and *bmp4* acts in concert with the directionality of cortical rotation imposed by sperm entry to set the initial dorsoventral molecular asymmetries that lead to embryonic axialization. In addition, it is also possible that this intrinsic egg bilaterality or latent dorsoventral prepattern is available as a backup mechanism for directionality of cortical rotation in case of failure of the mechanism directed by sperm entry, which occurs more frequently. The maternal bias in the distribution of molecules of known ventralizing properties before fertilization does not exclude that their spatial levels could be additionally controlled in relation to the fertilization-oriented cortical rotation. Nevertheless, our results imply that any control in the distribution of ventralizing molecules during or after fertilization might trigger mechanisms related to embryonic axialization that take their asymmetric prepattern in the unfertilized egg as a departure platform (Figure 8B).

Further experimental work is needed to study if the asymmetric maternal supply of molecules in an axis orthogonal to the animal-vegetal axis like those described here is providing ventralizing cues before fertilization. As discussed above, the expansion of a dorsal marker after maternal depletion of *notch1* mRNA in *Xenopus* (Mir et al., 2008) supports this hypothesis, at least for *notch1*. Although at lower levels than after zygotic genome activation, maternal *bmp4* transcripts were detected in *X. laevis* eggs by RNAseq (Session et al., 2016) and by ISH with a pattern that, although not commented by the authors, suggests an uneven distribution of transcripts in the animal hemisphere (Bell et al., 2003). In zebrafish embryos, maternal *bmp4* transcripts were detected in 2/8-cell or 64/128-cell by Northern blot and transcriptomics, respectively (Dick et al., 2000; Yang et al., 2013). In zebrafish, BMP4 signaling was proposed to be rather involved during late gastrulation in dorsoventral patterning, weakly contributing to ventralization, whereas BMP2b and BMP7a are mainly involved in early dorsoventral patterning (Stickney et al., 2007). Homozygous zebrafish embryos for a null *bmp4* allele (*bmp4* ^*st72*^) show a weak dorsalized phenotype in 73% of the cases, whereas heterozygous embryos look normal (Stickney et al., 2007), indicating that the null mutation results in a recessive dorsalized phenotype to which both, the maternal and the paternal allele, contribute. Strikingly, mating of adult females homozygous for this null *bmp4* allele with wild-type males produced 90% of heterozygous zebrafish embryos with a wild-type phenotype, while the remaining 10% showed a weak dorsalized phenotype. The authors concluded that there is no strict maternal requirement for BMP4 (Stickney et al., 2007), but in our view, their results do not rule out a subtle maternal effect with only 10% penetrance.

Regardless of the possible differences across species about gene contributions to early dorsoventral polarization, time-demanding loss-of-function experiments involving gene editing in *Xenopus* would help to illuminate if the asymmetric distribution of determinants in the egg have a significant impact on embryonic dorsoventral polarization. Functional experiments with the tools available for *Xenopus* until now could not discriminate between maternal and zygotic contributions. Addressing this issue would require generational intercrosses to study the effect of maternally/egg-derived mutant alleles or zygotically/sperm derived mutant alleles, as recently described for *wnt11b* loss-of-function in *X. laevis*, where this gene is present as singleton (Houston et al., 2022). A detailed analysis of the range and penetrance of phenotypes obtained should follow, because, for example, strong or weak phenotypes might be either zygotically- or maternally-related, or *vice versa*. This kind of analysis would be relatively more straightforward in the diploid species *Xenopus tropicalis* but might be complicated by the possibility that homozygous null mutations were embryonic lethal. This would require rescuing of homozygous embryos with injection of wild-type mRNA to obtain homozygous progenitors mutant for the gene under study, as previously done in the past for zebrafish mutants (Kishimoto et al., 1997; Dick et al., 2000).

### Possible mechanisms underlying Notch1 polarization

The development of the main embryonic axis in amphibians, fish, and birds is sensitive to gravity, but it is unlikely that gravity influences the specification of the animal-vegetal axis (Gerhart, 1980). Pigment always concentrates in the animal hemisphere, where the germinal vesicle is located, and yolk platelets in the vegetal region, where the mitochondrial clouds are first observed, regardless of the orientation of the oocytes in the follicle in relation to gravity. Therefore, it is thought that oocytes intrinsically build the animal-vegetal axis (Gerhart, 1980), whose development was better studied from sI to sVI and appears to be prefigured in oogonia, in the germline cyst (Kloc et al., 2004; Bilinski et al., 2017). Notably, Gerhart also discussed the observations of Wittek (1952) (quoted by Gerhart, 1980) in other amphibian species (*R. temporaria* and *T. alpestris*) where a precocious bilateral symmetry is apparent after oocyte maturation, with eccentric maturation spots in the pigmented animal hemisphere. This precocious bilateral symmetry could be traced back to a more lateral position of the germinal vesicle and the mitochondrial cloud at previtellogenic stages (Gerhart, 1980). After Gerhart discussed in 1980 all these important aspects, it was reported that the EMP’s eccentricity in *X. laevis* eggs might be related to the position held by the oocytes in the gravity field during maturation. Oocytes that were held during *in vitro* maturation with the GAP tipped 90° or 135° off axis in relation to the gravitational field showed maturation points with stronger eccentricity than oocytes that were held on axis, whose maturation points revealed very low or no eccentricity (Brown et al., 1994). Notably, in the process of *Xenopus* axialization, two periods of sensitivity to the centrifugal force were recognized during the first cell cycle (Black and Gerhart, 1985). Although the reason for this temporal change remains unexplained, it is striking that the centrifugal force could change the site of dorsalization from the centrifugal equatorial region before 40% of the cycle (i.e., before the onset of cortical rotation) to the opposite side (centripetal) between 40% and 70% of the cycle (i.e., during cortical rotation), when the only other variable was the position of the SEP, towards or away from the center of the rotor, as shown by these authors. Since the SEP usually marks the future ventral side, applying centrifugal force during the second period of sensitivity with the SEP towards the center of the rotor would completely reverse the directionality of cortical rotation and the displacement of maternal dorsal determinants from the vegetal cortex. A possible explanation for the first period of sensitivity might perhaps involve the relocation of ventralizing molecules already asymmetrically distributed before cortical rotation, related to the intrinsic bilateral symmetry or latent dorsoventral prepattern of the egg.

If the animal-vegetal axis were not influenced by gravity, the asymmetry of Notch1 immunofluorescence that we observe in an axis orthogonal to the animal-vegetal one, with variability of the immunofluorescence signal in the vegetal region during oogenesis might be related to gravity. Additional cues related to the resumption of meiosis during maturation, which appear to be also influenced by gravity, might refine this pattern. Although we have not attempted to demonstrate if Notch1 immunofluorescence correlates with the position of the EMP because this is technically more challenging, we have found that the asymmetrical *notch1* mRNA domain correlates with the EMP region.

Remarkably, it was earlier reported that *znf330 (Xnoa36)* and *α-spectrin* mRNAs and α-Spectrin protein are transiently segregated to a lateral half of mid-sI oocytes in a domain parallel to the animal-vegetal axis. This expression domain is opposite to the site from where the oocyte pends from the ovarian epithelium, which the authors called “hylum” (Vaccaro et al., 2010; Carotenuto and Tussellino, 2018) but would be better designated as “ovulation site” (Dumont and Brummett, 1978). Since these authors strictly adhered to the traditional view of dorsoventral axis formation being triggered after fertilization and did not contemplate the possibility of an intrinsic maternal bilateral symmetry which we thoroughly discuss in the present work, they doubted that the asymmetry they observed could anticipate a dorsoventral polarity (Vaccaro et al., 2010). Notwithstanding their interpretation, the results shown by these authors, which were overlooked in the literature, are in line with those described in the present work. They strongly support the idea of asymmetrical distribution of molecules along an axis orthogonal to the animal-vegetal one during *Xenopus* oogenesis.

Interestingly, Vaccaro et al. noticed that, apart from the mitochondrial cloud, which marks the future vegetal side of the oocyte, the only other asymmetry of the oocyte is relative to the position it occupies within the ovary (Vaccaro et al., 2010). The oocyte is completely surrounded by the follicular cells and the theca layer. The follicle is wrapped by the inner ovarian epithelium, except at the side facing the ovulation site, which instead contacts the outer ovarian epithelium (Dumont and Brummett, 1978). Both are squamous epithelia, but the outer one, lining the coelomic cavity, is composed of monociliated cells, whereas the inner one, lining the ovarian lumen, consists of a layer of nonciliated cells (Dumont and Brummett, 1978). It is known that primary cilia have a key role in the transduction of several morphogen pathways (Oh and Katsanis, 2012) and they are present in the outer ovarian epithelium in mammals (Teilmann and Christensen, 2005). Since the domain expressing *znf330 (Xnoa36)* and *α-spectrin* mRNAs and α-Spectrin protein in mid-sI oocytes is in the antipode of the ovulation site, the question arises if the developing oocyte is under the influence of differential signaling from these distinct epithelia which might polarize the oocyte in an axis different from the animal-vegetal one. If such differential signaling exists, it might contribute to developing the asymmetric Notch1 distribution we observed throughout oogenesis.

Further experimentation is needed to understand how the asymmetric Notch1 distribution described here arises and is maintained. Several aspects need to be considered in the future. For example, does this expression pattern bear any correlation with the ovulation site? Could it be influenced by gravity during oogenesis and oocyte maturation? Other aspects include examining the role of the cytoskeleton and whether mechanisms of transport, local stabilization or decay are involved. Cytoskeletal reorganization occurs during oogenesis (Carotenuto and Tussellino, 2018) and throughout the oocyte during maturation, the most conspicuous one related to germinal vesicle breakdown, meiotic spindle assembly and first polar body extrusion. During oocyte maturation, several important changes affect the three major filament systems of the cytoplasm (actin filaments, intermediate filaments, and microtubules) (Bement and Capco, 1990; Gard, 1992). Intriguingly, we notice that in unfertilized eggs, Gard observed an important variability regarding the orientation of the M2 spindle (which is assembled after polar body extrusion). In unfertilized eggs, the M2 spindle was either aligned with or transverse to the animal-vegetal axis. However, in *in vitro* matured oocytes, nearly all the M2 spindles became aligned with the animal-vegetal axis (Gard, 1992). We surmise that unfertilized eggs naturally matured in different orientations within the gravity field, whereas the *in vitro* matured oocytes mostly went through this process with the animal-vegetal axis aligned with the gravity field. Although this remains speculative, perhaps the grade of EMP eccentricity, which is influenced by gravity, is related to meiotic spindle orientation in this specialized asymmetric cell division that extrudes the first polar body. It remains to be elucidated if these processes are related with the Notch asymmetry we observed in this work. Experiments perturbing the cytoskeleton during oogenesis, oocyte maturation, and in eggs might clarify if cytoskeletal components are necessary for the establishment, refinement, or maintenance of the molecular asymmetries we describe here.

### Notch asymmetries during axis formation in bilaterians

We previously showed that Notch1 and β-Catenin proteins are asymmetrically and oppositely distributed during early embryogenesis in zebrafish as early as from the zygote stage (Castro Colabianchi et al., 2018). Now, we show that *notch1a* transcripts are also asymmetrically distributed in zebrafish embryos from the zygote stage. This conservation between fish and amphibians suggests that the control of the distribution of Notch1 on the dorsoventral axis is executed at the transcripts level, but we cannot rule out additional controls in the distribution of the protein for the maintenance of the asymmetry.

Our previous work showed that, in *X. laevis* embryos, Notch1 promotes β-Catenin destabilization through a non-canonical mechanism, independent of the DNA-binding protein RBPJ, which is the transcription factor that mediates canonical Notch, nuclear activity (Acosta et al., 2011). This non-canonical pathway explains part of Notch1 ventralizing activity (Castro Colabianchi et al., 2018) and was also described in *Drosophila* and in mammalian embryonic stem cells, where membrane-bound Notch associates to β-Catenin, promoting its degradation through the endocytic/lysosomal pathway (Hayward et al., 2005; Hayward et al., 2008; Sanders et al., 2009; Muñoz-Descalzo et al., 2010). Therefore, the early membrane-bound Notch1 asymmetry that we detected in this work before and immediately after cortical rotation might be relevant in destabilizing β-Catenin. More research is needed to demonstrate if Notch1 is already promoting β-Catenin degradation at these time points. In addition, we could detect ventral accumulation of Notch1 in nuclei at least from stage 7 in *X. laevis* embryos (i.e., before mid-blastula transition) (Castro Colabianchi et al., 2018).

Notably, a recent work described an asymmetry in the distribution of *notch1* transcripts in chicken embryos at early cleavage stages (Hwang et al., 2018). Although its functional significance was not studied, this points to the conservation of early *notch1* asymmetries in vertebrate embryos. Moreover, an antibody against the transcriptionally active Notch1 intracellular domain showed nuclear immunofluorescence in the outer cells but not in the inner cell mass of the mouse blastocyst, and Notch1 favors the specification of the trophectoderm lineage, disfavoring the inner cell mass lineage (Rayon et al., 2014). Therefore, this asymmetric Notch1 distribution is related to the earliest cell-fate choice in mammalian development, suggesting an active role in the polarity of the embryonic-abembryonic axis. More recently a reporter gene study revealed that an asymmetric Notch activity already exists as early as in the 4-cell stage mouse conceptus (Menchero et al., 2019). Symmetry breaking mechanisms are the subject of ongoing debate in mouse development. It was proposed that in most conceptus, the blastocyst’s axis of bilateral symmetry is parallel to the zygote’s animal-vegetal axis, whereas the blastocyst’s plane of bilateral symmetry and the embryonic-abembryonic axis are orthogonal to the first cleavage’s plane (Gardner, 2001; Johnson, 2009; Graham and Zernicka-Goetz, 2016). The body axes (dorsoventral and anterior-posterior) are definitively established in the period from implantation to pre-gastrulation. While the dorsoventral axis is indirectly inherited from the embryonic-abembryonic axis, the anterior-posterior axis of the embryo-fetus was proposed to correlate with the blastocyst’s axis of bilateral symmetry (Johnson, 2009; Zhang and Hiiragi, 2018).

In invertebrates like insects and cephalopods, eggs already show an obvious bilateral symmetry (Gardner, 1998; Houston, 2017). In *Drosophila melanogaster*, anterior-posterior and dorsoventral body axes are defined during oogenesis through the asymmetrical distribution of maternal determinants (Johnston and Nüsslein-Volhard, 1992; Stein and Stevens, 2014; Ma et al., 2016; Schüpbach, 2019). During *Drosophila* oogenesis, interactions between germline and somatic cells, involving Delta in the germline/oocyte and Notch in follicle cells participate in the establishment of the oocyte’s anterior-posterior axis, which determines the embryo’s anterior-posterior axis. Activation of the Notch pathway by Delta signaling from the oocyte is necessary for establishing the oocyte’s posterior pole (Ruohola et al., 1991; González-Reyes and St Johnston, 1998; López-Schier, 2003; Torres et al., 2003; Poulton and Deng, 2007; Sun et al., 2011; Schüpbach, 2019). In the nematode *Caenorhabditis elegans*, *glp-1* encodes one of the two *notch* orthologues present in this worm and is necessary for developing anterior cell fates. Maternal *glp-1* mRNA is uniformly distributed in zygotes and all blastomeres until the 8-cell stage. However, Glp-1 protein is asymmetrically distributed at the 2-cell stage, being localized in the blastomere fated to give rise to anterior tissues. This asymmetric distribution is controlled by translational repression and requires specific sequences in the 3′ UTR of *glp-1* mRNA (Evans et al., 1994).

Altogether, our findings, together with those from other animal models, point to the conservation of the involvement of the Notch pathway in establishing molecular asymmetries related to axis formation in Bilaterians.

## Data Availability

The original contributions presented in the study are included in the article/Supplementary Material, further inquiries can be directed to the corresponding author.
