# Supplementary material for: A maternal dorsoventral prepattern revealed by an asymmetric distribution of ventralizing molecules before fertilization in Xenopus laevis
Source: Front Cell Dev Biol. 2024 Mar 20;12:1365705. doi: 10.3389/fcell.2024.1365705 (PMC10987785; doi:10.3389/fcell.2024.1365705)
Supplement: Supplementary file 1 [file DataSheet1.PDF]

Supplementary Material

Figure S1

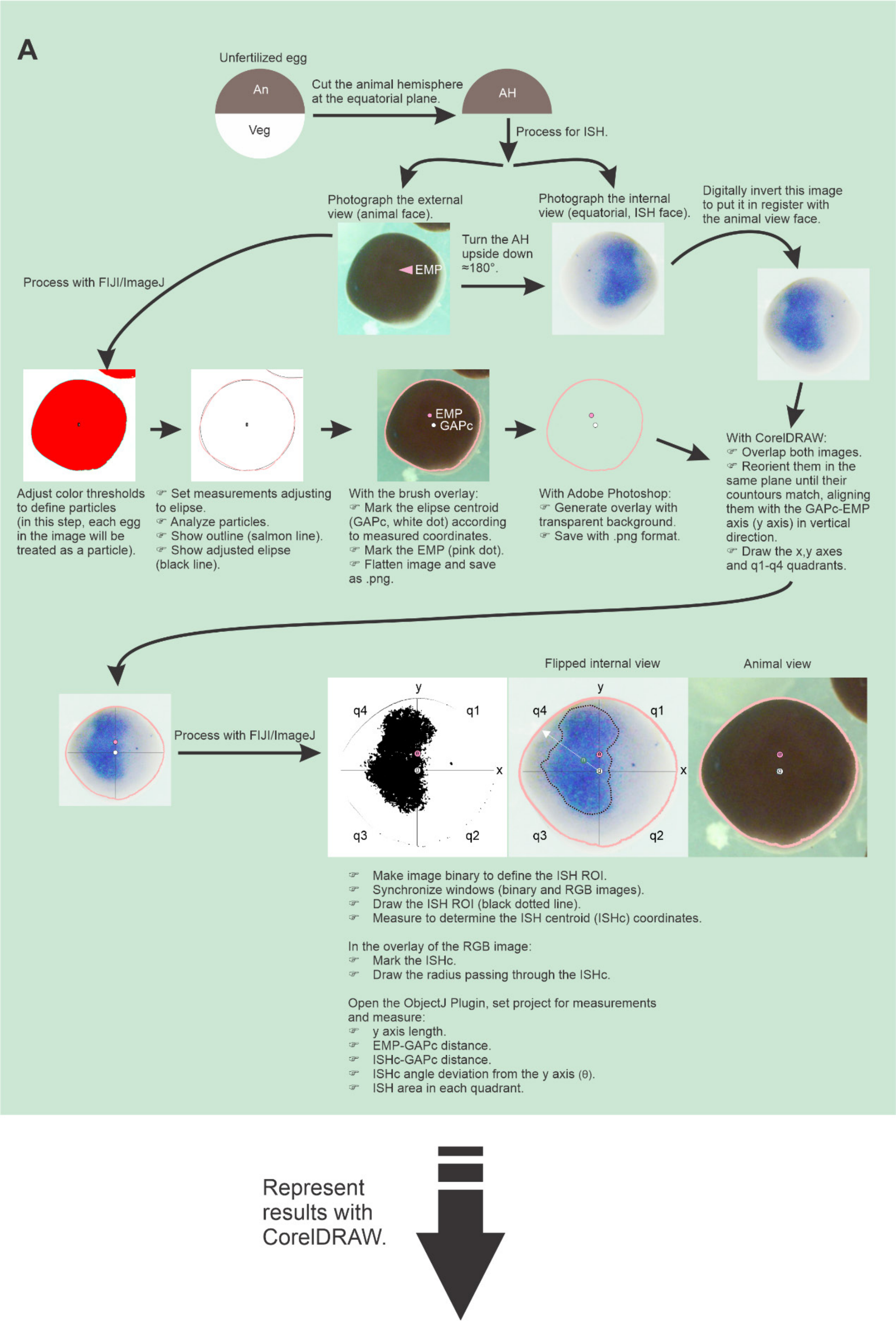
A
Unfertilized egg
An
Veg
Cut the animal hemisphere at the equatorial plane.
AH
Process for ISH.
Photograph the external view (animal face).
Photograph the internal view (equatorial, ISH face).
Digitally invert this image to put it in register with the animal view face.
EMP
Turn the AH upside down ≈180°.
Process with FIJI/ImageJ
EMP
GAPc
Adjust color thresholds to define particles (in this step, each egg in the image will be treated as a particle).
☞ Set measurements adjusting to elipse.
☞ Analyze particles.
☞ Show outline (salmon line).
☞ Show adjusted elipse (black line).
With the brush overlay:
☞ Mark the elipse centroid (GAPc, white dot) according to measured coordinates.
☞ Mark the EMP (pink dot).
☞ Flatten image and save as .png.
With Adobe Photoshop:
☞ Generate overlay with transparent background.
☞ Save with .png format.
With CorelDRAW:
☞ Overlap both images.
☞ Reorient them in the same plane until their countours match, aligning them with the GAPc-EMP axis (y axis) in vertical direction.
☞ Draw the x,y axes and q1-q4 quadrants.
Process with FIJI/ImageJ
Flipped internal view
Animal view
y
q4
q1
x
q3
q2
☞ Make image binary to define the ISH ROI.
☞ Synchronize windows (binary and RGB images).
☞ Draw the ISH ROI (black dotted line).
☞ Measure to determine the ISH centroid (ISHc) coordinates.
In the overlay of the RGB image:
☞ Mark the ISHc.
☞ Draw the radius passing through the ISHc.
Open the ObjectJ Plugin, set project for measurements and measure:
☞ y axis length.
☞ EMP-GAPc distance.
☞ ISHc-GAPc distance.
☞ ISHc angle deviation from the y axis (θ).
☞ ISH area in each quadrant.
Represent results with CorelDRAW.

B

♀	Egg #	ϴ ISHc angle (°)	$rEGD_{diam}$ (% of YL)	$rEGD_{rad}$ (% of CR)	$rIGD_{diam}$ (% of YL)	$rIGD_{rad}$ (% of CR)
81	3	-54,22	11%	21%	13%	25%

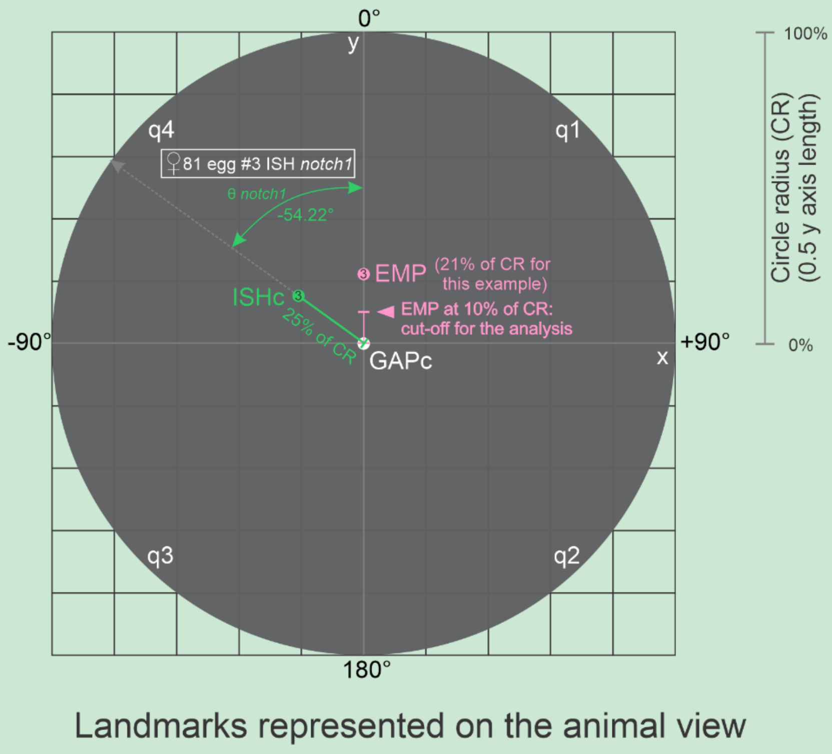

Figure S1: Morphometric analysis procedure for ISH domains. (A) Example of the analysis for *notch1* ISH with egg #3 from female #81. See project for measurements with the ObjectJ plugin in Supplementary Methods below this figure. Measurements for this egg are included in Table S4. (B) Graphic representation of measurements in a polar coordinates template. See more details for the whole procedure in the main text. AH, animal hemisphere.

Supplementary Methods

Measurement project for the ObjectJ Plugin

1. In the Objects box tool, we defined the following objects to draw in the image linked to the project:

Images | Objects | Columns | Qualifiers | ObjectJ Results | Show in Explorer

☑ Show Object Number ☑ Composite Objects
☐ 3D Objects ☐ Selective Item Visibility

Item Name	Item Shape	Clones	Marker Type	Line Type	Item Color
ISH centroid	Point	1	Cross	1 pt	Yellow
EMP	Point	1	Cross	1 pt	Red
GAP centroid	Point	1	Cross	1 pt	Cyan
Y ext intersect	Point	1	Diamond	1 pt	Blue
G-ISH ext intersect	Point	1	Diamond	1 pt	Blue
Y axis	Line	1	Dot	1 pt	Dark Gray
ISH area Q1	Polygon	1	Dot	1 pt	Black
ISH area Q2	Polygon	1	Dot	1 pt	Black
ISH area Q3	Polygon	1	Dot	1 pt	Black
ISH area Q4	Polygon	1	Dot	1 pt	Black

- Y ext intersect: the point of the y axis intersecting the egg's outline.
- Y-axis: a line encompassing the entire y-axis length.
- G-ISH ext intersect: the point of the GAPc-ISHc radius intersecting the egg's outline.

2. In the Columns box tool, we defined the following operations:

Operation	1st Operand Item type	Clone#	Point#	2nd Operand Item type	Clone#	Point#	3rd Operand Item type	Clone#	Point#
Angle	G-ISH ext inters...	1	1	GAP centroid	1	1	Y ext intersect	1	1
Distance	EMP	1	1	GAP centroid	1	1			
Distance	ISH centroid	1	1	GAP centroid	1	1			
Distance	EMP	1	1	ISH centroid	1	1			

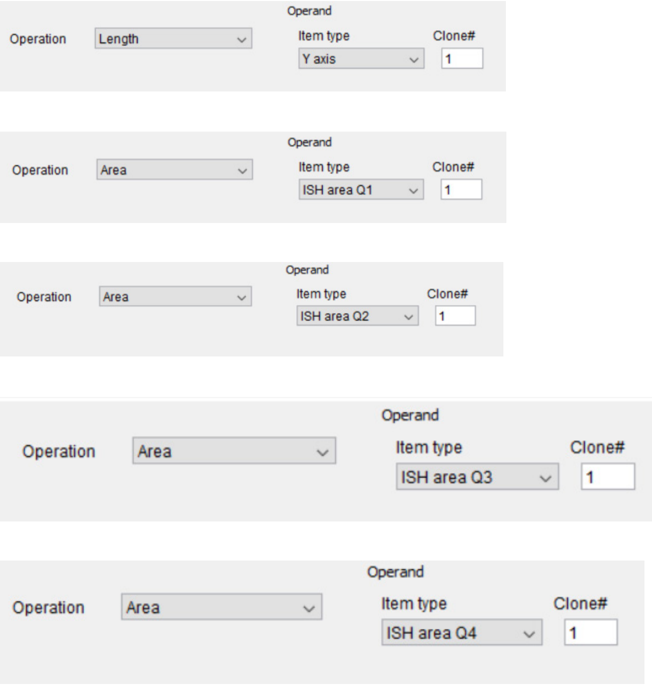

Screen capture of the procedure, with objects in the list (left side of the figure) marked up to the ISH q1 area, just about proceeding to demarcate the ISH q2 area:

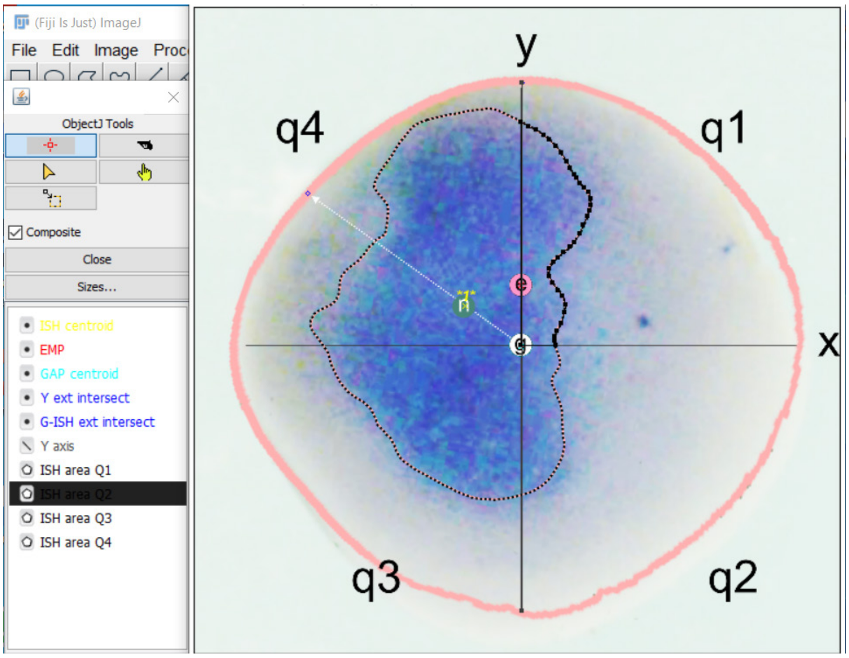

Figure S2.

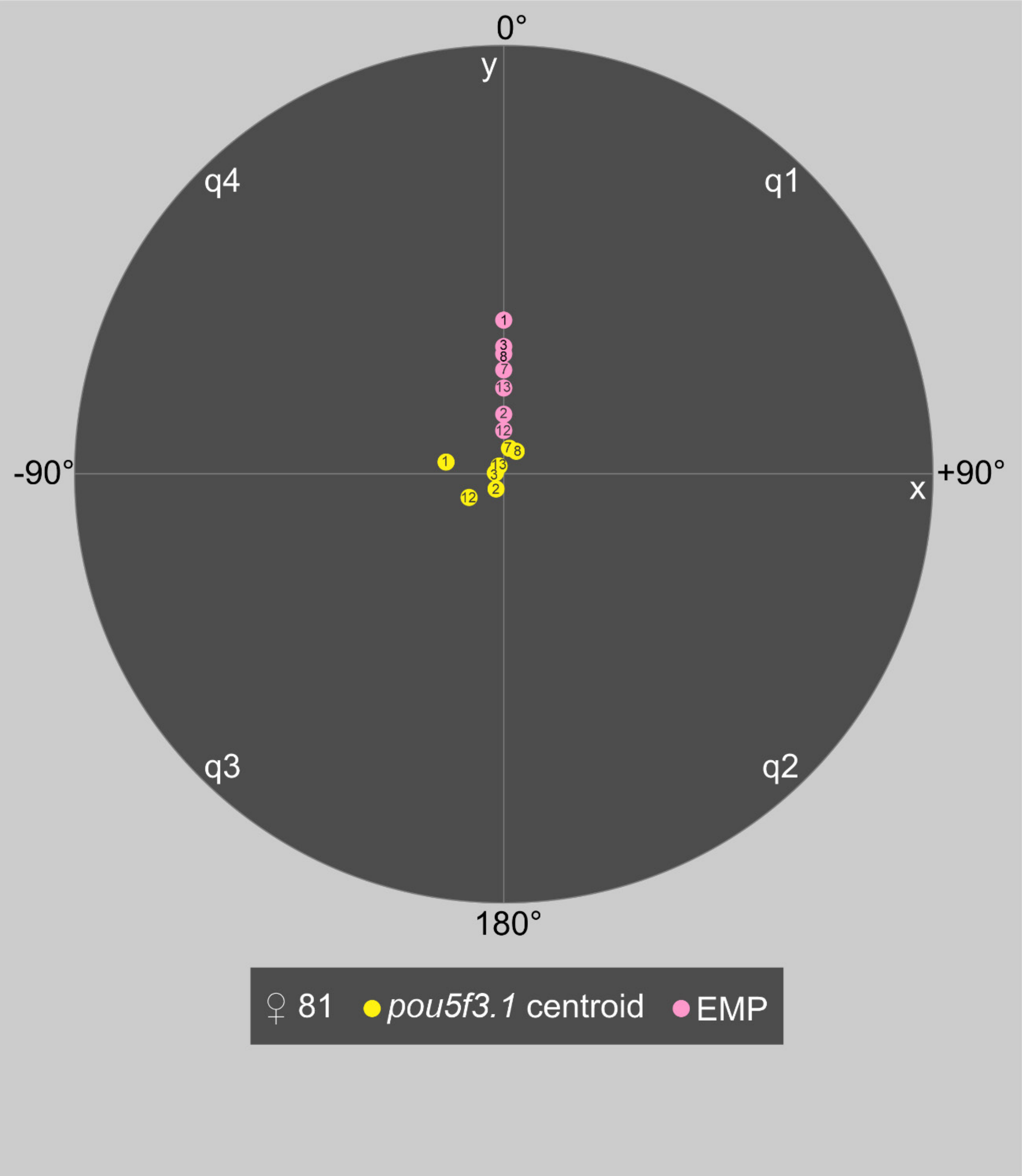

Figure S2. Polar coordinates graphic showing the spatial distribution of EMP and ISH centroids for *pou5f3.1* for eggs from female #81. The numbers within the circles indicate the individual egg #. The graphic was constructed according to the results shown in Table S4.

Figure S3.

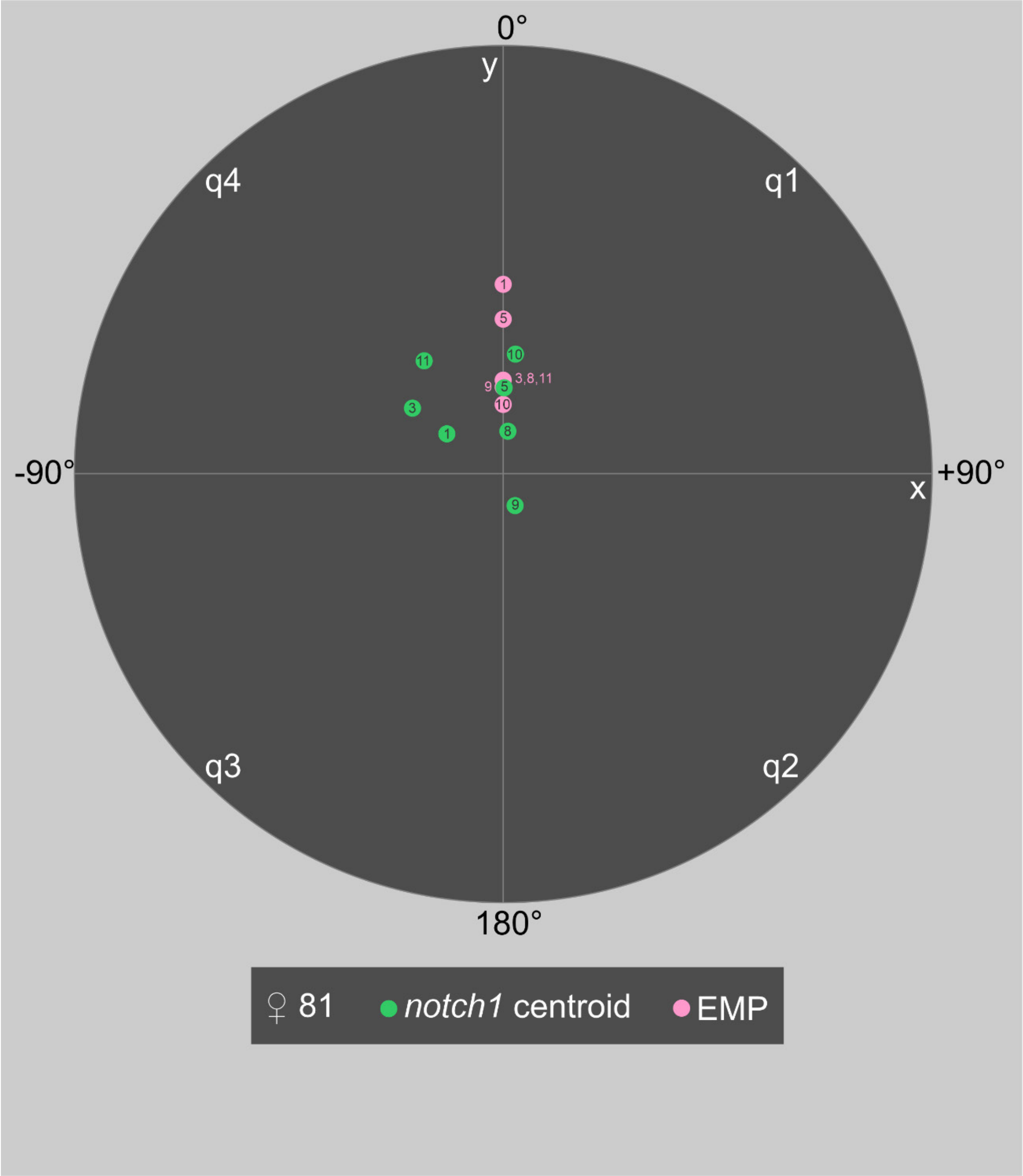

Figure S3. Polar coordinates graphic showing the spatial distribution of EMP and ISH centroids for *notch1* for eggs from female #81. The numbers within or beside the circles indicate the individual egg #. The graphic was constructed according to the results shown in Table S4.

Figure S4.

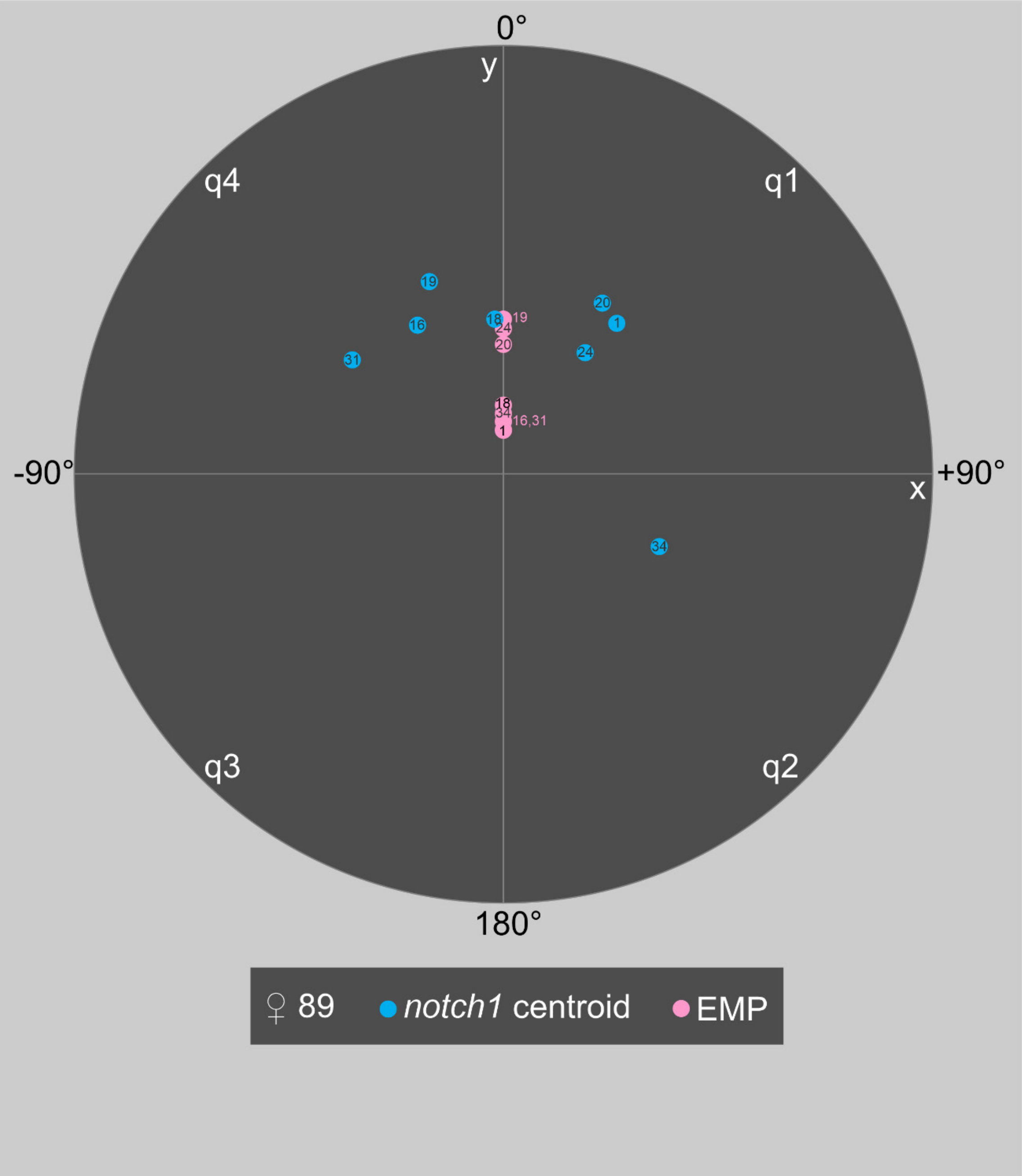

Figure S4. Polar coordinates graphic showing the spatial distribution of EMP and ISH centroids for *notch1* for eggs from female #89. The numbers within or beside the circles indicate the individual egg #. The graphic was constructed according to the results shown in Table S4.

Movie S1.

Image transition from Fig. S2, S3, and S4 to graphic composite shown in Fig. 5F.

Figure S5.

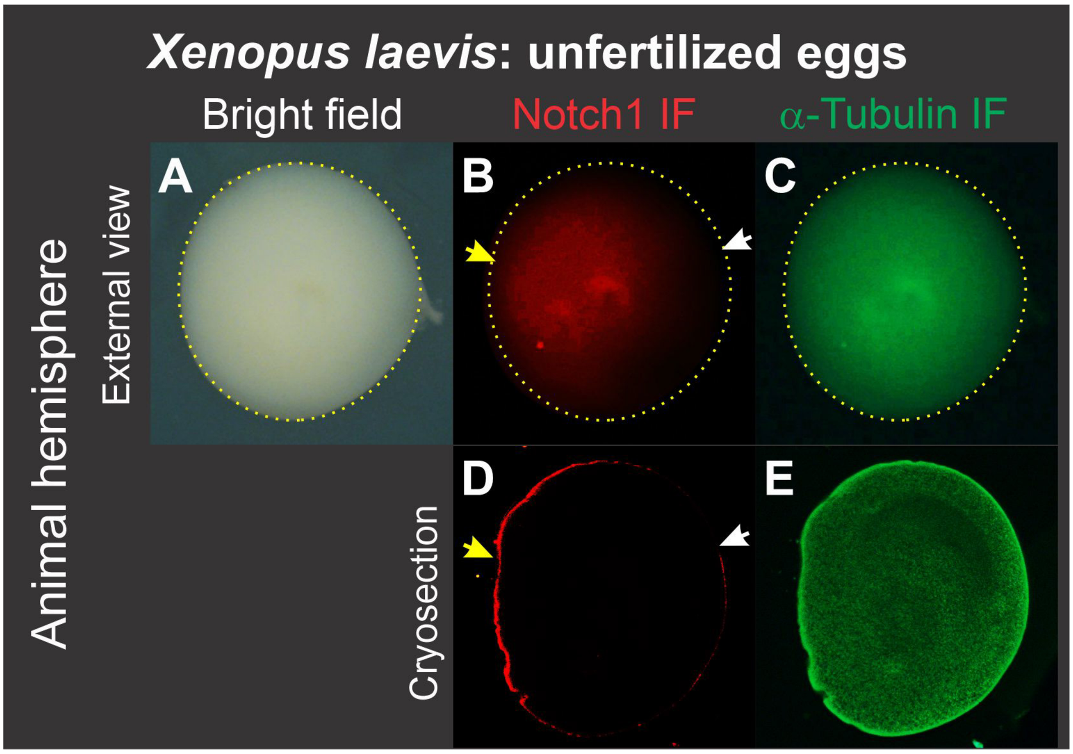

Figure S5. Asymmetric distribution of Notch1 protein in the animal hemisphere of unfertilized *X. laevis* eggs. Pigmented animal hemispheres were dissected by cutting them away through the equatorial plane, bleached, and directly processed for whole-mount immunofluorescence (A-C) or cryosections (D,E) followed by immunofluorescence for Notch1 (red fluorescence; B,D) and α-Tubulin (green fluorescence; C,E) as ubiquitous reference protein. Yellow and white arrows respectively point to the highest and lowest Notch1 immunofluorescence signal, indicating that Notch1 protein is asymmetrically distributed in the animal hemisphere in unfertilized eggs.

Supplementary Tables

X. laevis zygotes	Distribution	IF<br>Notch1 protein	ISH 1<br>*notch1* mRNA	ISH 2<br>*notch1* mRNA	TOTAL<br>*notch1* mRNA
**Before cortical rotation**	Asymmetric	19/19<br>(Fig. 2A-D)	3/3	-	3/3<br>(Fig. 2E-E')
	Symmetric	0/19	0/3	-	0/3
**End of cortical rotation**	Asymmetric	10/10<br>(not shown) (*)	11/11	5/5	16/16<br>(Fig. 2F-F') (**)
	Symmetric	0/10	0/11	0/5	0/16

Supplementary Table 1. Notch1 protein and mRNA are asymmetrically distributed in the animal hemisphere of *X. laevis* zygotes before the onset of cortical rotation. Albino embryos at s1 were fixed before or at the end of cortical rotation. They were processed for immunofluorescence (IF) for Notch1 protein combined with ISH for *gdf1 (vg1)* mRNA or double ISH for *notch1* and *gdf1 (vg1)* mRNAs. As *gdf1* transcripts are uniformly distributed in the vegetal cortex, this marker was used as a spatial reference for orienting the zygotes in their animal-vegetal axis. Since in double ISH, *gdf1* was revealed with BCIP in the first place, the signal was often lost during the steps for revealing the second probe (*notch1*), and those zygotes were not considered for scoring. Only zygotes with *gdf1* staining were considered for scoring the number of them showing the asymmetric distribution of Notch1 protein or mRNA in the animal hemisphere. We never observed uniform distribution of Notch1 protein or mRNA in zygotes. ISH1 and ISH2 represent batches of eggs from different females. As expected from our previous findings (Castro Colabianchi et al., 2018), Notch1 protein (*) and *notch1* mRNA (**) were asymmetrically distributed in the animal hemisphere at the end of cortical rotation.

X. laevis unfertilized eggs Immunofluorescence	R1 Notch1	R2 Notch1	R3 Notch1/ α-tubulin	CRYO1 Notch1/ GAPDH	CRYO2 Notch1/ α-tubulin	ALBINO IF Notch1/ ISH *wnt11b*
	Pigmented eggs					Albino eggs
Notch1 Asymmetric distribution	52/54	15/16	29/29	17/17	4/5	8/9
Total	96/99, N=3			21/22, N=2		8/9, N=2
**TOTAL**	**125/130 (96%), N=6**					

Supplementary Table 2. Asymmetric distribution of Notch1 protein throughout the animal hemisphere of unfertilized *X. laevis* eggs. R1 to R3, CRYO1, CRYO2, and ALBINO correspond to independent biological replicates (eggs obtained from different females). In R1 to R3, CRYO1, and CRYO2, pigmented animal hemispheres were dissected from eggs by cutting them away through the equatorial plane, bleached and directly processed for immunofluorescence (IF) (R1 to R3) for Notch1 alone or combined with immunofluorescence for α-tubulin, or processed for cryosections (CRYO1, CRYO2), followed by immunofluorescence for Notch1 combined with immunofluorescence for α-tubulin or GAPDH as ubiquitous reference proteins. Whole albino eggs were processed for ISH of *wnt11b* mRNA combined with Notch1 immunofluorescence. The orientation of albino eggs in the animal-vegetal axis was verified by the location of *wnt11b* mRNA as a reference, which is uniformly distributed in the vegetal hemisphere in eggs. Only albino eggs with *wnt11b* staining were considered for scoring the number of them showing the asymmetric distribution of Notch1 protein in the animal hemisphere. Values indicate the number of eggs with asymmetric distribution of Notch1 protein throughout the animal hemisphere in relation to the total number of eggs analyzed. N, number of biological replicates.

X. laevis unfertilized eggs																
ISH	*notch1*						*bmp4*		*dll1*		*hes4*		*pou5f3.1*			
	Single ISH		Double ISH (with *gdf1*)		Double ISH (with *pou5f3.1*)		Single ISH		Single ISH		Single ISH		Single ISH		Double ISH (with *notch1*)	
	Animal hemisphere (pigmented)		Whole embryo (albino)		Whole embryo (albino)		Animal hemisphere (pigmented)		Animal hemisphere (pigmented)		Animal hemisphere (pigmented)		Animal hemisphere (pigmented)		Whole embryo (albino)	
	♀	Asymmetric	♀	Asymmetric	♀	Asymmetric	♀	Asymmetric	♀	Asymmetric	♀	Asymmetric (weak)	♀	Central	♀	Central
	#80	7/8	#111	5/5	A	10/10	#81	38/38	#20	18/19	#81	11/12	#81	12/13	A	10/10
	#81	13/14	#121	11/11			#89	34/40								
	#89	14/14	#122	8/8												
Total	34/36, N=3		24/24, N=3		10/10, N=1								12/13, N=1		10/10, N=1	
TOTAL	**Asymmetric *notch1***						**Asymmetric *bmp4***		**Asymmetric *dll1***		**Asymmetric *hes4* (weak)**		**Central *pou5f3.1***			
	**68/70 (97%) N=7**						**72/78 (92%) N=2**		**18/19 (95%) N=1**		**11/12 (92%) N=1**		**22/23 (96%) N=2**			

Supplementary Table 3. Distribution of *notch1* mRNA and other transcripts throughout the animal hemisphere of unfertilized *X. laevis* eggs. Pigmented eggs were cut through the equatorial plane. animal hemispheres were processed for ISH for the indicated markers. Whole albino eggs were processed for double ISH for *notch1* mRNA and *gdf1 (vg1)* mRNA or *pou5f3.1* mRNA. For double *notch1/gdf1* ISH, the orientation of albino eggs in the animal-vegetal axis was verified by the location of *gdf1* mRNA as a reference, which is uniformly distributed in the vegetal cortex. Only albino eggs with *gdf1* or *pou5f3.1* staining were considered for scoring the distribution of *notch1* mRNA in the animal hemisphere. Values indicate the number of eggs with the asymmetric or central distribution of the indicated marker throughout the animal hemisphere in relation to the total number of eggs analyzed. N indicates the number of biological replicates (number of independent females employed to obtain eggs).

Supplementary Table 4. Results from the morphometric analysis of *notch1* and *pou5f3.1* distribution in unfertilized *X. laevis* eggs. See Table 1 in the main text for definitions and abbreviations of parameters.

See the accompanying Excel file.

X. laevis oocytes Notch1 IF	sI asymmetric	sII asymmetric	sIII asymmetric	sIV asymmetric	sV asymmetric	sVI asymmetric	Total asymmetric
IF A	12/12	9/9	6/6	6/6	6/6	6/6	29/29
IF B	7/7	3/3	7/7	6/7	4/4	1/1	28/29
IF A'	11/12	10/10	6/8	6/7	9/9	8/8	50/54
**Total asymmetric /stage**	30/31	22/22	19/21	19/20	19/19	9/9	**118/122**

Supplementary Table 5. Notch1 protein is asymmetrically distributed during *X. laevis* oogenesis. Defolliculated oocytes from pigmented females were fixed, bleached, and processed for combined immunofluorescence (IF) for Notch1 protein and ISH for *wnt11b* mRNA as a reference marker of the vegetal pole to orient the oocytes in their animal-vegetal axis. Only oocytes with *wnt11b* staining were considered for scoring the number of them showing the asymmetric distribution of Notch1 protein. IF A and IF A' represent two independent Notch1 immunofluorescence/ *wnt11b* ISH assays of oocytes from female A. IF B represents another Notch1 immunofluorescence/ *wnt11b* ISH assay of oocytes from an independent female. Oocytes were classified according to Dumont (1972) (Dumont, 1972).

Zebrafish *notch1a* mRNA distribution (ISH)										
**Stage**	**R1**		**R2**		**R3**		**R4**		**R1+R2+R3+R4**	
	**A**	**S**	**A**	**S**	**A**	**S**	**A**	**S**	**A**	**S**
1c					6	0	22	0	28	0
2c					2	0	24	0	26	0
4c	3	0	2	0	5	0	21	0	31	0
8c	5	0			1	0	15	0	21	0
16c	6	0					4	0	10	0
32c	3	0					12	0	15	0
64c			1	0					1	0
Sphere			2	0					2	0
**Total**	**17**	**0**	**5**	**0**	**14**	**0**	**98**	**0**	**134 (100%)**	**0 (0%)**

Supplementary Table 6. Zebrafish *notch1a* mRNA is asymmetrically distributed along the animal hemisphere during early embryogenesis. The spatial expression of *notch1a* transcripts was analyzed by ISH from the 1-cell stage (zygote) until the sphere stage. R1 to R4 are biological replicates from four independent mating pairs. A, asymmetric distribution; S, symmetric distribution.

References

Castro Colabianchi, A. M., Revinski, D. R., Encinas, P. I., Baez, M. V., Monti, R. J., Rodríguez Abinal, M., et al. (2018). Notch1 is asymmetrically distributed from the beginning of embryogenesis and controls the ventral center. *Development* 145, dev159368. doi: 10.1242/dev.159368.

Dumont, J. N. (1972). Oogenesis in Xenopus laevis (Daudin). I. Stages of oocyte development in laboratory maintained animals. *J. Morphol.* 136, 153–179. doi: 10.1002/jmor.1051360203.
